# GPSD: a hybrid learning framework for the prediction of phosphatase-specific dephosphorylation sites

**DOI:** 10.1093/bib/bbae694

**Published:** 2025-01-02

**Authors:** Cheng Han, Shanshan Fu, Miaomiao Chen, Yujie Gou, Dan Liu, Chi Zhang, Xinhe Huang, Leming Xiao, Miaoying Zhao, Jiayi Zhang, Qiang Xiao, Di Peng, Yu Xue

**Affiliations:** Department of Bioinformatics and Systems Biology, MOE Key Laboratory of Molecular Biophysics, Hubei Bioinformatics and Molecular Imaging Key Laboratory, Center for Artificial Intelligence Biology, College of Life Science and Technology, Huazhong University of Science and Technology, Luoyu Road 1037, Wuhan, Hubei 430074, China; Department of Bioinformatics and Systems Biology, MOE Key Laboratory of Molecular Biophysics, Hubei Bioinformatics and Molecular Imaging Key Laboratory, Center for Artificial Intelligence Biology, College of Life Science and Technology, Huazhong University of Science and Technology, Luoyu Road 1037, Wuhan, Hubei 430074, China; Department of Bioinformatics and Systems Biology, MOE Key Laboratory of Molecular Biophysics, Hubei Bioinformatics and Molecular Imaging Key Laboratory, Center for Artificial Intelligence Biology, College of Life Science and Technology, Huazhong University of Science and Technology, Luoyu Road 1037, Wuhan, Hubei 430074, China; Department of Bioinformatics and Systems Biology, MOE Key Laboratory of Molecular Biophysics, Hubei Bioinformatics and Molecular Imaging Key Laboratory, Center for Artificial Intelligence Biology, College of Life Science and Technology, Huazhong University of Science and Technology, Luoyu Road 1037, Wuhan, Hubei 430074, China; Department of Bioinformatics and Systems Biology, MOE Key Laboratory of Molecular Biophysics, Hubei Bioinformatics and Molecular Imaging Key Laboratory, Center for Artificial Intelligence Biology, College of Life Science and Technology, Huazhong University of Science and Technology, Luoyu Road 1037, Wuhan, Hubei 430074, China; Department of Bioinformatics and Systems Biology, MOE Key Laboratory of Molecular Biophysics, Hubei Bioinformatics and Molecular Imaging Key Laboratory, Center for Artificial Intelligence Biology, College of Life Science and Technology, Huazhong University of Science and Technology, Luoyu Road 1037, Wuhan, Hubei 430074, China; Department of Bioinformatics and Systems Biology, MOE Key Laboratory of Molecular Biophysics, Hubei Bioinformatics and Molecular Imaging Key Laboratory, Center for Artificial Intelligence Biology, College of Life Science and Technology, Huazhong University of Science and Technology, Luoyu Road 1037, Wuhan, Hubei 430074, China; Department of Bioinformatics and Systems Biology, MOE Key Laboratory of Molecular Biophysics, Hubei Bioinformatics and Molecular Imaging Key Laboratory, Center for Artificial Intelligence Biology, College of Life Science and Technology, Huazhong University of Science and Technology, Luoyu Road 1037, Wuhan, Hubei 430074, China; Department of Bioinformatics and Systems Biology, MOE Key Laboratory of Molecular Biophysics, Hubei Bioinformatics and Molecular Imaging Key Laboratory, Center for Artificial Intelligence Biology, College of Life Science and Technology, Huazhong University of Science and Technology, Luoyu Road 1037, Wuhan, Hubei 430074, China; Department of Bioinformatics and Systems Biology, MOE Key Laboratory of Molecular Biophysics, Hubei Bioinformatics and Molecular Imaging Key Laboratory, Center for Artificial Intelligence Biology, College of Life Science and Technology, Huazhong University of Science and Technology, Luoyu Road 1037, Wuhan, Hubei 430074, China; School of Artificial Intelligence and Automation, Huazhong University of Science and Technology, Luoyu Road 1037, Wuhan, Hubei 430074, China; Department of Bioinformatics and Systems Biology, MOE Key Laboratory of Molecular Biophysics, Hubei Bioinformatics and Molecular Imaging Key Laboratory, Center for Artificial Intelligence Biology, College of Life Science and Technology, Huazhong University of Science and Technology, Luoyu Road 1037, Wuhan, Hubei 430074, China; Department of Bioinformatics and Systems Biology, MOE Key Laboratory of Molecular Biophysics, Hubei Bioinformatics and Molecular Imaging Key Laboratory, Center for Artificial Intelligence Biology, College of Life Science and Technology, Huazhong University of Science and Technology, Luoyu Road 1037, Wuhan, Hubei 430074, China

**Keywords:** posttranslational modification, dephosphorylation site, protein phosphatase, machine learning, transfer learning

## Abstract

Protein phosphorylation is dynamically and reversibly regulated by protein kinases and protein phosphatases, and plays an essential role in orchestrating a wide range of biological processes. Although a number of tools have been developed for predicting kinase-specific phosphorylation sites (p-sites), computational prediction of phosphatase-specific dephosphorylation sites remains to be a great challenge. In this study, we manually curated 4393 experimentally identified site-specific phosphatase–substrate relationships for 3463 dephosphorylation sites occurring on phosphoserine, phosphothreonine, and/or phosphotyrosine residues, from the literature and public databases. Then, we developed a hybrid learning framework, the group-based prediction system for the prediction of phosphatase-specific dephosphorylation sites (GPSD). For model training, we integrated 10 types of sequence features and utilized three types of machine learning methods, including penalized logistic regression, deep neural networks, and transformer neural networks. First, a pretrained model was constructed using 561 416 nonredundant p-sites and then fine-tuned to generate computational models for predicting general dephosphorylation sites. In addition, 103 individual phosphatase-specific predictors were constructed via transfer learning and meta-learning. For site prediction, one or multiple protein sequences in FASTA format could be inputted, and the prediction results will be shown together with additional annotations, such as protein–protein interactions, structural information, and disorder propensity. The online service of GPSD is freely available at https://gpsd.biocuckoo.cn/. We believe that GPSD can serve as a valuable tool for further analysis of dephosphorylation.

## Introduction

Reversible protein phosphorylation is one of the most important posttranslational modifications (PTMs) and determines the functional dynamics of targeted substrates, such as protein activity, localization, interactions, and stability [1]. Phosphorylation is involved in regulating a broad spectrum of biological processes, such as cellular metabolism, transcription, and cell division [2, 3]. Mechanistically, protein kinases (PKs) serve as writers to transfer one or multiple phosphoryl groups for the modification of substrates, whereas protein phosphatases (PPs) function as erasers to specifically dephosphorylate substrates by hydrolysing phosphate ester bonds for the removal of phosphoryl groups [4, 5]. In eukaryotes, phosphorylation and dephosphorylation mainly occur on three types of phosphorylatable amino acid residues, including serine (S), threonine (T), and tyrosine (Y) residues [6]. Furthermore, these two types of catalytic enzymes dynamically control the equilibrium of phosphorylation and dephosphorylation, which determines the steady state of protein phosphorylation levels *in vivo* [7, 8]. The balance of reversible phosphorylation is responsible for sustaining the cellular homeostasis under normal physiological conditions [9, 10]. In particular, aberrant PP activity results in inadequate or excessive protein phosphorylation to be implicated in numerous human diseases, including cancer, neurodegenerative disorders, and diabetes [7, 11, 12]. Thus, the identification of phosphatase-specific targets and site-specific phosphatase–substrate relationships (ssPSRs) is fundamental for understanding the regulatory mechanisms of dephosphorylation.

The detection of phosphatase-specific targets and dephosphorylation sites via traditional biochemical methods, such as *in vitro* phosphorylation assay and immunoblotting, is usually low throughput (LTP), labour intensive, and time consuming. In recent years, the advancement of various high-throughput (HTP) technologies, including protein chips and tandem mass spectrometry (MS/MS), has facilitated the discovery of dephosphorylation sites [13–19]. For example, Hoermann *et al*. combined a substrate phosphorylation peptide library and a phosphoproteomic approach to analyse dephosphorylation events involving phosphoserine (pS) and phosphothreonine (pT) residues, which are demodified by the catalytic subunits of PPs PP1 and PP2A [14]. In addition to experimental assays, *in silico* analyses of dephosphorylation sites have also been employed. To date, several tools have adopted conventional machine learning methods to detect dephosphorylation events on phosphotyrosine (pY) residues for a small number of tyrosine PPs, including protein tyrosine phosphatase 1B (PTP1B) and the Src homology 2 (SH2) domain-containing PPs SHP-1 and SHP-2 [20–22]. For example, Wu *et al*. used the *k*-nearest neighbour algorithm and sequence features to predict pY sites of substrates specifically demodified by PTP1B, SHP-1, and SHP-2 [20]. Later, Wang *et al*. separately utilized the Group-based Prediction System (GPS) method and CKSAAP-DEPHOS, which combined a support vector machine (SVM) with the composition of *k*-spaced amino acid pairs (CKSAAP) approach, to establish two predictors for the analyses of dephosphorylation sites [21]. Moreover, Jia *et al*. integrated a bi-profile Bayes feature extraction technique and an SVM to predict dephosphorylation sites that are specific for three tyrosine PPs [22]. Recently, Chaudhari *et al*. used a bidirectional long short-term memory (Bi-LSTM) method for the development of DTL-DephosSite to predict general dephosphorylation sites on pS, pT, and pY residues [23]. The number of PPs needs to be expanded for dephosphorylation prediction. Previously, we used 490 762 nonredundant eukaryotic p-sites to pretrain a general phosphorylation model, which markedly increased the accuracy for the prediction of kinase-specific p-sites [24]. It is not known whether such a pretraining strategy would facilitate the computational detection of dephosphorylation sites in eukaryotes.

In this study, we first collected 4393 reported ssPSRs for 3463 dephosphorylation sites occurring on pS, pT, and/or pY residues of 1833 protein substrates, as well as their corresponding 106 upstream PPs, from the literature and public databases (Supplementary Table S1). Then, we developed a computational tool, the group-based prediction system for the prediction of phosphatase-specific dephosphorylation sites (GPSD). For model training, 10 types of sequence features were used, and three machine learning methods, including penalized logistic regression (PLR), deep neural networks (DNNs), and transformer neural networks (TNNs), were integrated into a hybrid learning framework. Compared with a previously reported tool DTL-DephosSite [23], GPSD exhibited a highly comparative accuracy for predicting general dephosphorylation sites. By combining transfer learning and meta-learning, we further fine-tuned 103 individual models for predicting phosphatase-specific dephosphorylation sites, using 4267 reported ssPSRs. For convenience, an online service of GPSD was developed. Overall, we anticipate that GPSD could serve as a useful tool for further analysis of dephosphorylation.

## Methods

### The algorithm of GPSD

In this study, we developed a three-step framework for predicting phosphatase-specific dephosphorylation sites in eukaryotes. First, general phosphorylation models were pretrained and fine-tuned to construct the models for predicting general dephosphorylation sites. Then, individual phosphatase-specific predictors were further fine-tuned from the general dephosphorylation models. The details on data collection and preparation, as well as sequence feature encoding, are provided in Supplementary methods. The implementation of GPSD is presented as below.

To pretrain general phosphorylation models, we first defined a p-site peptide PSP (30,30) as a phosphorylatable residue flanked by 30 upstream residues and 30 downstream residues, as previously described [24]. The PSP (30,30) items around known p-sites were regarded as positive data, whereas the PSP (30,30) items from other non-phosphorylatable S/T or Y residues were taken as negative data. Next, we used 10 types of sequence features to encode PSP (30,30) items (Supplementary methods). For each feature, PLR and DNN were separately used to train a model. Using one-hot encoding, two additional models were trained by Bidirectional Encoder Representations from Transformers (BERT) and Generative Pre-trained Transformer (GPT), respectively [25–27].

For each PSP (30,30) item, 20 prediction scores were individually produced by each of the 10 DNN models (*D*_1_, *D*_2_, *D*_3_, …, *D*_10_) and 10 PLR models (*P*_1_, *P*_2_, *P*_3_, …, *P*_10_). Two additional scores, *B* and *G*, were produced by BERT- and GPT-based models, respectively. These scores were represented as a 22-dimensional vector as follows:


$$ V=\left({D}_1,{D}_2,{D}_3,\dots, {D}_{10},{P}_1,{P}_2,{P}_3,\dots, {P}_{10},B,G\right) $$

Then, the vector *V* was used as the secondary feature, and a new PLR model was trained based on this vector to obtain a final score.

For fine-tuning general dephosphorylation models, the pretrained parameters of general phosphorylation models were unchanged. Similarly, the dephosphorylatable PSP (30,30) items were regarded as positive data, whereas other non-dephosphorylatable PSP (30,30) items in the same substrates were taken as negative data.

To further fine-tune the phosphatase-specific models, reported ssPSRs were hierarchically classified according to the levels of PP groups, PP families, and individual PPs [28]. For each PP cluster that included ≥30 dephosphorylation sites, its corresponding model was implemented directly using transfer learning, and *n*-fold cross-validations were conducted to evaluate its performance. For other PP clusters that contained <30 dephosphorylation sites, Model-Agnostic Meta-Learning (MAML) [29, 30], a widely used meta-learning strategy, was adopted for model fine-tuning. For each PP cluster, the negative PSP (30,30) items were randomly resampled, with a positive-versus-negative ratio of 1:10 per time. In total, 20 independent iterations were performed for fine-tuning the DNN models and TNN models. The leave-one-out validation was performed to test the performance. The training procedures were interactively conducted until the accuracy of predictive models was not increased any longer.

The PLR model was implemented in scikit-learn v1.0.2 (https://scikit-learn.org/stable/), with the ridge (L2) penalty. The ‘lbfgs’ solver was adopted for parameter optimization. For comparison, three additional machine learning methods, including SVM, random forest (RF), and Gaussian Naïve Bayes (GNB), were also implemented in scikit-learn v1.0.2. The DNN model was implemented in Keras 2.4.3 (http://github.com/fchollet/keras) with the TensorFlow 2.4.1 backend, as well as the BERT- and GPT-based models. Details on implementation of DNNs and TNNs were present in Supplementary methods. For the DNN framework, the optimized parameters, including the number of neurons, dropout ratio, and learning rate, are provided in Supplementary Table S2. A computer with an NVIDIA GeForce RTX 2060 GPU, a Genuine Intel CPU @ 3.60 GHz CPU, and 64 GB of RAM was used for training the computational models.

## Results

### Development of a hybrid learning framework for the prediction of dephosphorylation sites

Since dephosphorylation only occurs at p-sites, we first pretrained general phosphorylation models. The training dataset contained 561 416 nonredundant known p-sites in 82 468 proteins derived from three public databases, including EPSD [31], dbPTM [32], and PhosphoSitePlus [33]. From iLearnPlus, a machine learning platform for analysing biological sequences [34], we obtained 66 informative features to encode protein sequences. To assess the usefulness of each of the 66 features, PLR was first used for model training, and 9 sequence features were ultimately selected for their superior performance values. For encoding each PSP (30,30) item, nine sequence features were used together with the GPS feature [24]. Then, PLR and DNNs were separately used to construct a model using each of the 10 features. Using one-hot encoding, we further took two frameworks of TNNs, including BERT and GPT, to learn the contextual information [25–27]. The prediction scores of the 22 models were taken as the secondary features and further integrated by PLR to output a single predictive score.

**Figure 1:**
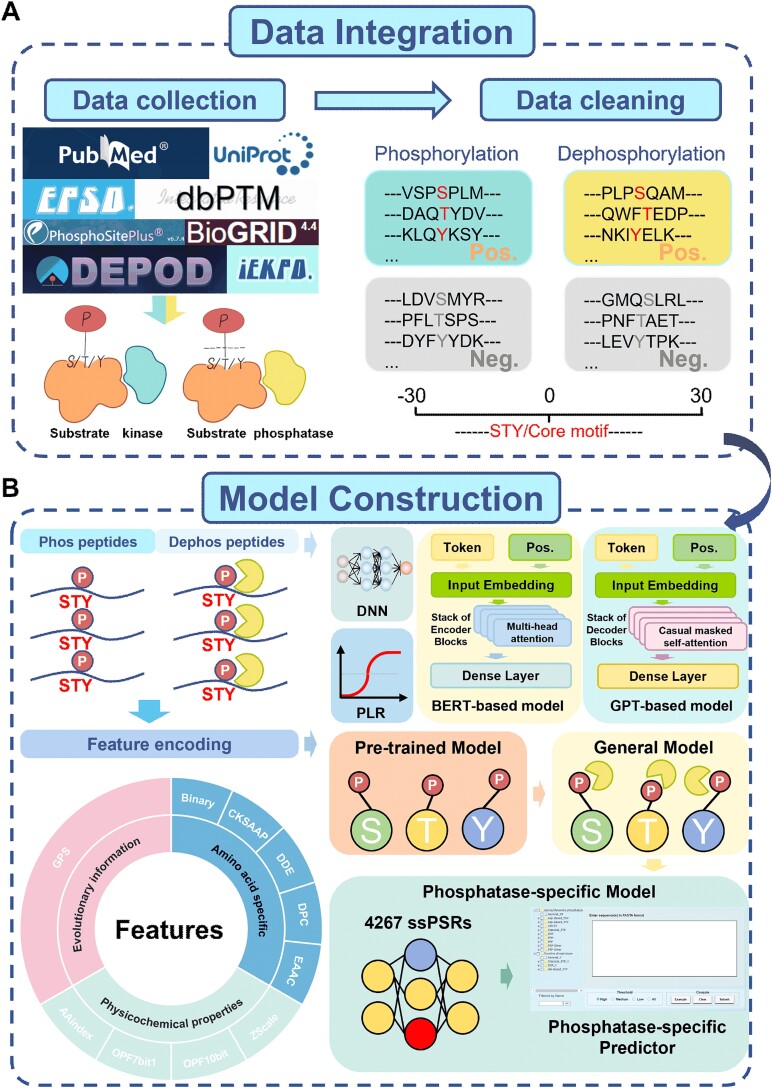
The whole procedure for the development of GPSD. (A) Data collection and preparation of nonredundant p-sites and dephosphorylation sites from the literature and public databases. (B) The model construction of GPSD for predicting eukaryotic dephosphorylation sites. In total, 10 types of sequence feature and 3 machine learning approaches, including DNNs, PLR, and TNNs, were integrated into a hybrid learning framework for model training. The strategy of pretraining followed by fine-tuning was adopted to develop phosphatase-specific predictors.

Next, the pretrained models for predicting general phosphorylation were fine-tuned for constructing general dephosphorylation predictors. The benchmark dataset contained 3304 nonredundant dephosphorylation sites in 1765 proteins collected from the literature and two public databases, DEPOD [35] and dbPTM [32] (Supplementary Table S1). Then, the general dephosphorylation models were further fine-tuned to construct 103 phosphatase-specific predictors, utilizing 4267 ssPSRs of 3304 dephosphorylation sites in 1765 phosphatase-specific substrates (Fig. 1A, Supplementary Table S1). PP clusters with fewer than three dephosphorylation sites were not included for model implementation. In particular, considering the limited numbers of experimentally validated ssPSRs for the majority of PPs, a meta-learning method, MAML [29, 30], was employed to enhance the robustness and accuracy of predictive models trained with fewer than 30 phosphatase-specific sites. Using transfer learning and meta-learning, a total of 103 phosphatase-specific predictors were generated. Finally, a website server was provided to be freely accessible at https://gpsd.biocuckoo.cn/ (Fig. 1B).

### Performance evaluation and comparison

**Figure 2:**
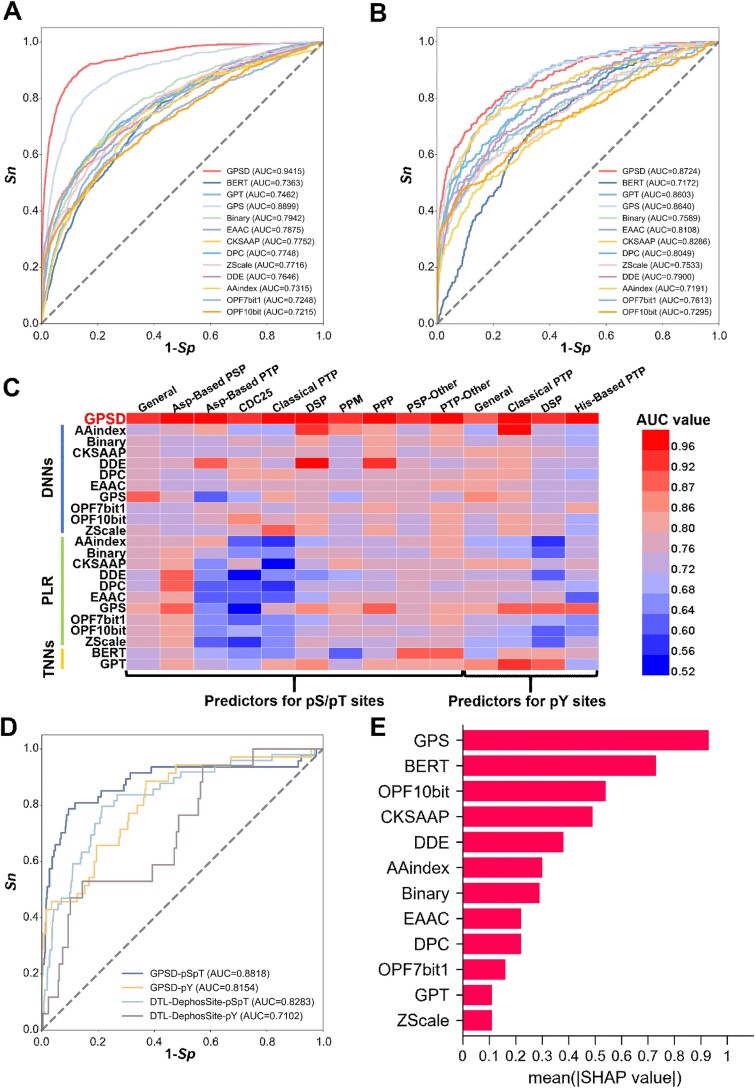
Performance evaluation and comparison of the GPSD. (A) Performance evaluation of the predictors of pS/pT dephosphorylation sites via various features and algorithms. The 10 models were individually trained with each of the 10 sequence features by DNNs. Using one-hot encoding, two models were trained by BERT and GPT, respectively. The GPSD predictor was trained via combining three machine learning methods and 10 sequence features. The ROC curves and AUC values are presented separately for the 13 predictive models. (B) Performance evaluation of the predictors of pY dephosphorylation sites via various algorithms and features. Similar to the pS/pT dephosphorylation site predictors, 13 predictors were shown to evaluate the model performance. (C) the AUC values of dephosphorylation predictors using each of 10 sequence features and 3 machine learning methods, including DNNs, PLR, and TNNs. (D) Performance comparison of the general model of GPSD with the previously reported tool via an independent test dataset. (E) Contribution of the 10 types of sequence features and the contextual information captured by TNNs for predicting dephosphorylation pS/pT site by measuring the SHAP score.

Using the 10-fold cross-validation, the performance of each feature was individually assessed. The area under the curve (AUC) values for predicting pS/pT dephosphorylation sites ranged from 0.7215 (OPF_10bit in the DNN model) to 0.8899 (GPS in the DNN model) (Fig. 2A and Supplementary Fig. S1A). Similarly, for the prediction of dephosphorylation at pY residues, the AUC values ranged from 0.6869 (AAindex in the PLR model) to 0.8640 (GPS in the DNN model) (Fig. 2B and Supplementary Fig. S1B). The receiver operating characteristic (ROC) curves revealed that the sequence feature encoded by the GPS method consistently had greater predictive accuracy for dephosphorylation sites than the other nine remaining sequence features did. Using one-hot encoding, BERT and GPT were adopted to extract contextual information from phosphorylatable and dephosphorylatable peptides, respectively. Our results showed that the general models of predicting pS/pT dephosphorylation using BERT- and GPT-based architecture received the AUC values of 0.7363 and 0.7462, respectively (Fig. 2A). For the prediction of pY dephosphorylation sites, the AUC values of BERT- and GPT-based models were 0.7172 and 0.8603, respectively (Fig. 2B).

Next, we evaluated the performance of each sequence feature using PLR, DNNs, or TNNs. Taken the GPS feature as an example, the general predictor of pS/pT dephosphorylation trained by DNNs achieved the highest AUC value of 0.8899, while the pY dephosphorylation model of dual-specificity phosphatase (DSP) trained with PLR obtained a higher AUC value of 0.9109 compared to other features trained by other machine learning methods (Fig. 2C). In addition, for the other phosphoserine phosphatase (PSP-Other) family, the AUC value of BERT-based predictor was 0.8895, greater than other features using DNNs or PLR (Fig. 2C). Thus, different machine learning methods exhibited differential capabilities in learning various sequence features. After the integration of all 10 features and 3 machine learning approaches, the 10-fold cross-validation AUC value reached 0.9415 for predicting pS/pT dephosphorylation, and the AUC value of the predictive model for dephosphorylation sites at pY residues was 0.8724 (Fig. 2A–C, and Supplementary Fig. S1C, Supplementary Table S3). The confusion matrices also supported the performance of GPSD for predicting general dephosphorylation sites (Supplementary Fig. S2A). Besides PLR, we further used SVM, RF, and GNB for feature integration. The similar 10-fold cross-validation values supported the efficiency of PLR (Supplementary Fig. S1D, E). In this regard, our analyses indicated the hybrid learning framework was helpful for improving the prediction accuracy.

In addition, 4-, 6-, and 8-fold cross-validations were performed to further evaluate the general dephosphorylation predictors (Supplementary Fig. S1F, G). The AUC values were similar to the 10-fold cross-validation results, supporting the robustness of models in GPSD. Furthermore, for 25 phosphatase-specific models curated with ≥30 experimentally validated sites, 4-, 6-, 8-, and 10-fold cross-validations were performed, as well as confusion matrices under the 10-fold cross-validation (Supplementary Figs. S2B and S3). Our analyses showed that the AUC values ranged from 0.9207 to 1.000, implying the superior performance of GPSD for predicting phosphatase-specific dephosphorylation sites. Moreover, we compared the performance values of models for predicting dephosphorylation sites, with or without the pretraining models of general phosphorylation (Supplementary Fig. S1D, E). The predictors fine-tuned from pretraining models had higher AUC values than did the models without pretraining (Supplementary Fig. S1D, E).

Next, we compared the performance of GPSD for predicting general dephosphorylation, to a previously published tool, DTL-DephosSite (Supplementary Table S4) [23]. We used an independent dataset not for training, including a total of 159 dephosphorylation sites. GPSD showed AUC values of 0.8818 and 0.8154 for predicting pS/pT and pY dephosphorylation sites, respectively, much higher than DTL-DephosSite (Fig. 2D). Furthermore, we investigated the contribution of each of the 10 sequence features to dephosphorylation prediction, and a widely used method, SHapley Additive exPlanation (SHAP), was employed for model interpretations (Fig. 2E and Supplementary Fig. S1H) [36]. The results revealed that all sequence features were informative for predicting dephosphorylation sites, and the GPS feature achieved the highest scores, indicating the importance of sequence similarity in the prediction of modified sites. Moreover, we observed that the contextual information captured by BERT had a higher contribution for predicting pS/pT dephosphorylation sites (Fig. 2E), whereas GPT-based model had a higher contribution for predicting pY dephosphorylation sites (Supplementary Fig. S1H). The contextual information learnt by either BERT or GPT was useful for improving the prediction performance. Taken together, our results demonstrated that combining 10 sequence features and 3 machine learning methods facilitated the prediction of dephosphorylation sites in eukaryotes.

### Usage of the GPSD web server

**Figure 3:**
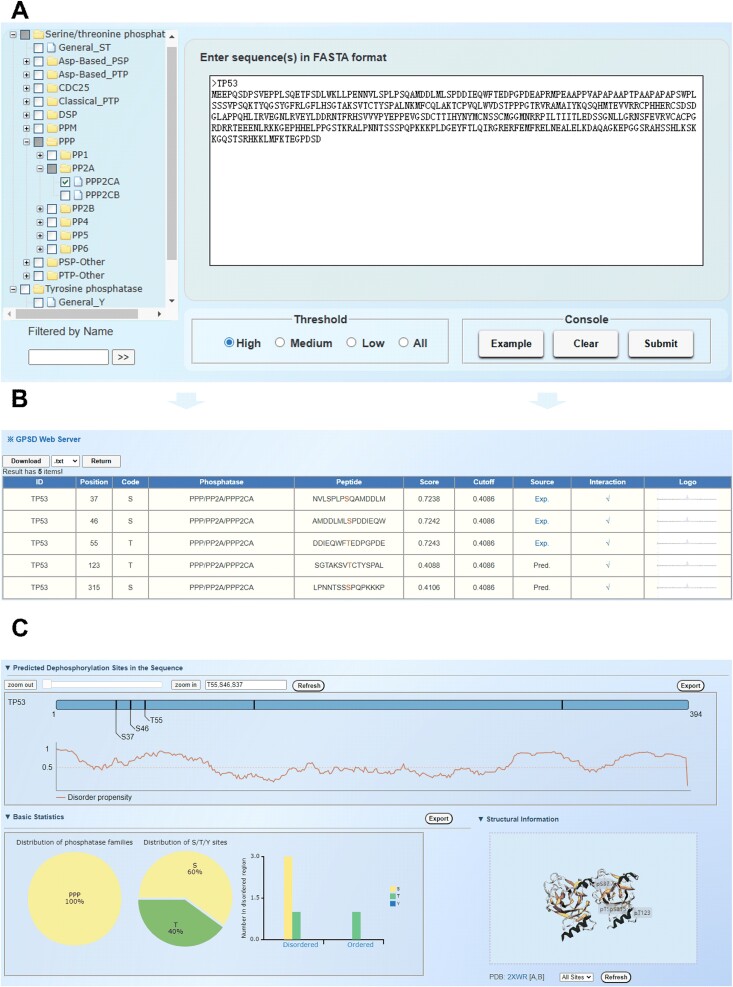
Usage of the GPSD web server. (A) The sequence submission interface. Users can input protein sequences in FASTA format and select from three different thresholds for predicting phosphatase-specific dephosphorylation sites. (B) Presentation of the prediction results with the example. The tabular list includes the positions of the dephosphorylation sites, prediction scores, cutoff values, identification via experimental or computational methods, PPI information, and iceLogo results. (C) Annotations of the prediction results. The number of dephosphorylation sites predicted by GPSD is presented, along with the locations of these sites illustrated in a 3D structure derived from the PDB database. Additionally, the disorder score of the dephosphorylation sites was calculated.

For convenience, we developed a user-friendly web server of GPSD to computationally predict general and phosphatase-specific dephosphorylation sites (Fig. 3). Users can submit single or multiple protein sequences in FASTA format via the prediction interface, with adjustable thresholds (Fig. 3A). The results table displays information such as ‘ID’, ‘Position’, ‘Phosphatase’, ‘Peptide’, ‘Score’, and ‘Source’ (Fig. 3B). Clicking ‘Exp’ in the ‘Source’ column links to PubMed evidence, if available, while the ‘Interaction’ column indicates interaction data from the BioGrid database [37]. For each PP cluster, the sequence logo of the DSP (30,30) items generated by iceLogo software is displayed in the ‘Logo’ column [38]. All the columns can be sorted by clicking the title (Fig. 3B). By default, the top three dephosphorylation sites with the highest predicted scores, along with the disorder propensity score for each residue predicted by IUPred [39], are shown in the protein sequence diagram. We also conducted basic statistical analyses on the distribution and number of disordered regions within the selected PP family. Additionally, the 3D structure of the substrate with the predicted dephosphorylation site can be presented via 3Dmol.js (Fig. 3C). Prediction results are downloadable in .txt, .csv, .tsv, and .xlsx formats, and images can be exported as .png files. For convenience, we provided a video tutorial with 1′42″ for a step-by-step usage of the online service of GPSD.

### Motif analysis of dephosphorylation sites

**Figure 4:**
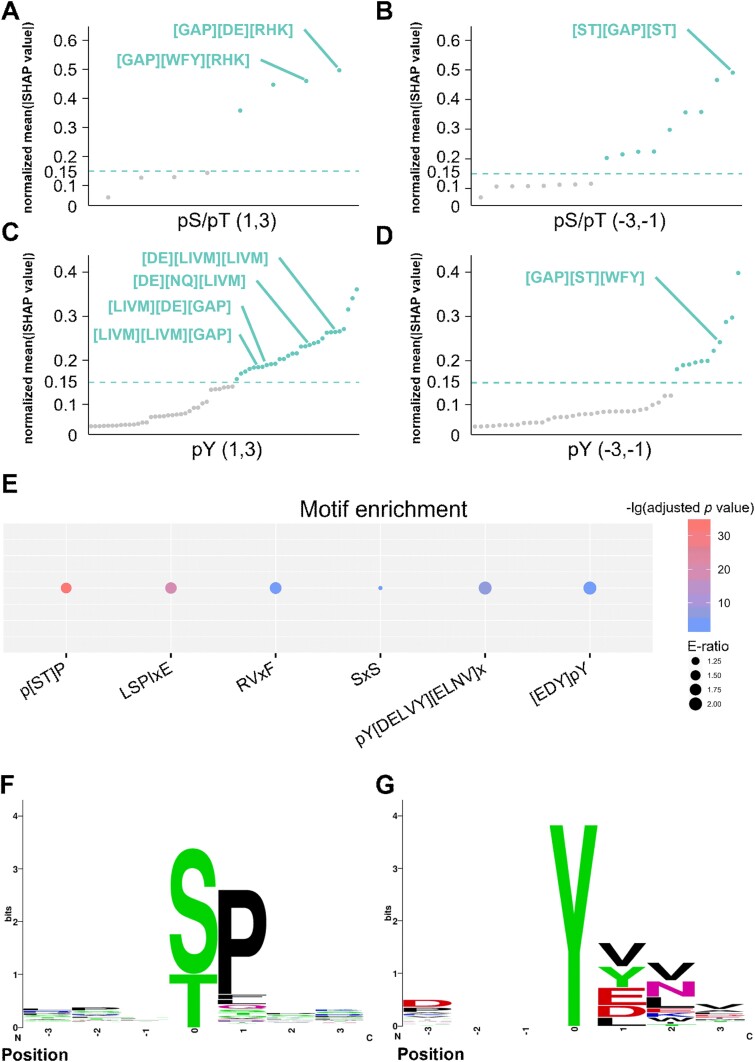
Motif analysis of dephosphorylation sites. (A) PCCs between the frequency of each peptide and the SHAP value at (1, 3) location adjacent to the pS/pT dephosphorylation site. (B) PCCs between the frequency of each peptide and the SHAP value at (−3, −1) location adjacent to the pS/pT dephosphorylation site. (C) PCCs between the frequency of each peptide and the SHAP value at (1, 3) location adjacent to the pY dephosphorylation site. (D) PCCs between the frequency of each peptide and the SHAP value at (−3, −1) location adjacent to the pY dephosphorylation site. (E) Enrichment analysis of experimentally validated classical dephosphorylation motifs, including the LSPIxE motif, RVxF motif, and [ST]P. (F, G) Sequence logos that conform to the experimentally validated classical dephosphorylation motifs. Motif sequences on pS/pT phosphatase-associated substrates (F) and motif sequences on tyrosine-related substrates (G).

We utilized the SHAP [36] method to analyse the motif sequences that are potentially essential for protein dephosphorylation. After dividing and calculating the DSP(3,3) for the pS/pT sites and pY sites, we constructed the frequency matrix and SHAP value matrix of the peptide at each position. To understand the effects of adjacent peptides on dephosphorylation modification, we calculated the Pearson correlation coefficients (PCCs) between the frequency of each peptide and the SHAP value at each location (Supplementary Fig. S4). The use of a threshold with an absolute PCC value >0.2 as a cutoff indicated that 8, 17, 63, and 44 peptides in the 4 positions from upstream to downstream might be important for protein modification at the pS, pT, or pY residues. In addition, we calculated the average SHAP score for each peptide after *Z*-score normalization. A normalized average SHAP value >0.15 was used as the threshold to determine the protein peptides that might play an essential role in dephosphorylation modification. After the analyses, 4, 9, 29, and 11 short peptides were reserved at each position (Fig. 4A–D and Supplementary Table S5).

According to previous studies on dephosphorylation sites, several consensus motif sequences have been detected, including LSPIxE [40, 41], RVxF [41, 42], and p[ST]P [43, 44] (p[STY] represents the dephosphorylation site and x represents any amino acid residue). In this study, we explored the sequence motifs of dephosphorylation sites and evaluated the reliability of our manually curated datasets for predictive model training. Here, we first compiled 55 dephosphorylation motif sequences and their corresponding PPs through collecting and curating the literature (Supplementary Table S5). Using these 55 known motifs as a benchmark dataset, we analysed the protein peptides extracted from our analysis results and evaluated which motifs were significantly enriched. For dephosphorylation occurring at pS/pT residues, approximately half of the amino acids detected at the +1 position of pS/pT sites in phosphorylated proteomes treated with PP1 and PP2A are proline residues [14]. Our findings revealed that the peptides starting with [GAP] at position (1, 3) of the pS/pT sites received higher scores (Fig. 4A). Moreover, our enrichment analysis demonstrated that the p[ST]P motif was significantly overrepresented (Fig. 4E–F). In addition, the known sequence motif SxS [45] was significantly enriched (Fig. 4E). Consistently, the peptides beginning with p[ST] at the position (−3, −1) of the pS/pT residues obtained the highest score (Fig. 4B). With respect to the dephosphorylation of tyrosine residues, our results demonstrated that the sequence motifs pY[DELVY][ELNV]x [46] and [EDY]pY [47–49] were significantly overrepresented (Fig. 4E and G). Moreover, the peptides starting with [DE] and [LIVM] at position (1, 3) of the pY residue had a higher score, and the sequence ending with [WFY] at position (−3, −1) of the pY site received a higher score (Fig. 4C and D). In addition, we discovered that classical motifs, including LSPIxE and RVxF, were also enriched (Fig. 4E) [40–42]. Our analyses revealed the reliability of the modification sites used for the training of the predictive models in our curated datasets.

### Analysis of phosphatase-specific dephosphorylation sites

**Figure 5:**
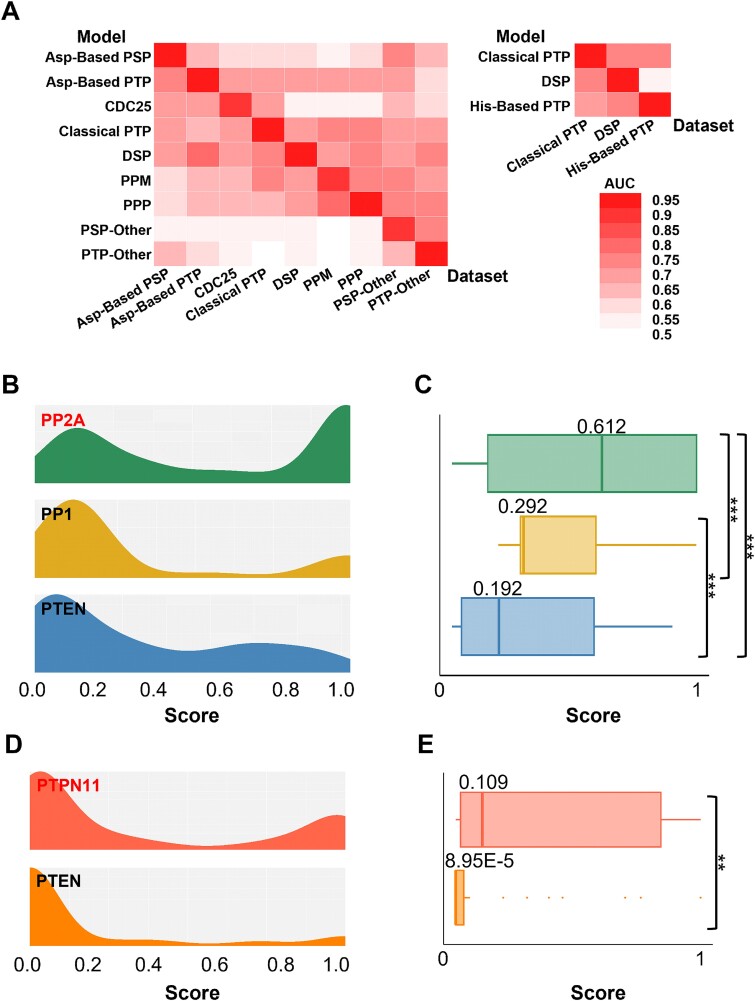
Specificity analysis of PP dephosphorylation site predictors. (A) Specificity analysis of pS/pT dephosphorylation site predictors and pY dephosphorylation site predictors on the basis of family classification via the AUC value. (B, C) The density (B) and box (C) charts show the distributions of pS/pT dephosphorylation site predictors specific for PP2A, PP1, and PTEN in the PP2A dataset. (D, E) The density (D) and box (E) charts show the distributions of pY dephosphorylation site predictors specific for PTPN11 and PTEN in the PTPN11 dataset.

To evaluate the specificity of dephosphorylation prediction via individual predicative models, we conducted an analysis of the phosphatase-specific dephosphorylation sites. For verification of prediction accuracy, nine PPs-specific predictors for the dephosphorylation of pS/pT sites and three PPs-specific predictors for the dephosphorylation of pY sites were employed at the group level of PPs. In the evaluation, each predictor was used to predict the positive datasets of dephosphorylation sites from the other models and to validate the accuracy of the phosphatase-specific modification sites. Compared with the AUC values generated from other models, each predictive model showed greater accuracy in the prediction of their corresponding positive datasets of modified sites (Fig. 5A).

Next, to further assess prediction accuracy at the single PP level, we selected predictors for pS/pT dephosphorylation specific to PP1, PP2A, and PTEN, as well as predictors for pY dephosphorylation specific to PTPN11 (SHP-2) and PTEN. For each selected PP, positive datasets of dephosphorylation sites were used to measure prediction specificity. First, PP2A-specific sites were adopted to evaluate the accuracy of the three predictors, revealing significantly lower prediction scores for PP1 and PTEN compared to PP2A using 10-fold cross-validation (Fig. 5B, C). Similarly, analysis of predictors specific to PTPN11 and PTEN showed significantly higher prediction scores for PTPN11 on PTPN11-positive datasets (Fig. 5D, E). We also evaluated predictors on other phosphatase-specific dephosphorylation site datasets (Supplementary Fig. S5), confirming the prediction specificity of these models. In summary, our findings demonstrated that the PP-specific predictors are able to specifically recognize and computationally identify the modified sites of targeted substrates.

### Prediction of potential cancer-associated dephosphorylation events

**Figure 6:**
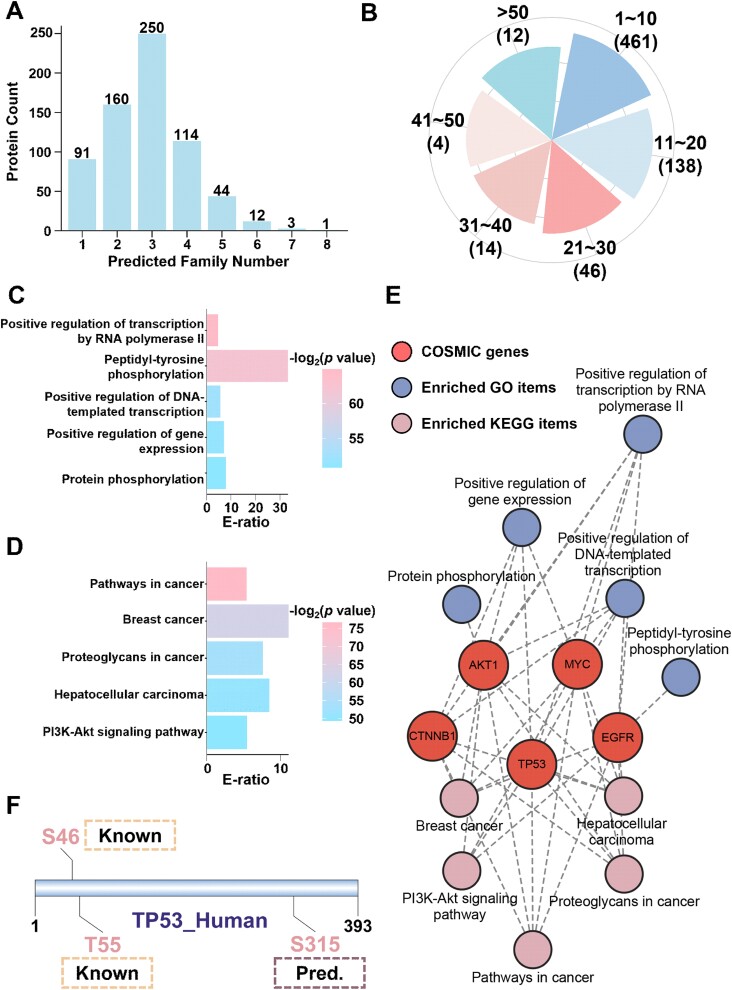
Cancer-associated dephosphorylation events predicted by GPSD. (A) Distribution of cancer proteins predicted to be dephosphorylated by 12 PP families with GPSD. (B) Distribution of the number of predicted dephosphorylation sites in cancer proteins. (C) GO-based enrichment analysis of 174 cancer proteins predicted to be dephosphorylated by at least 4 PP families. (D) KEGG-based enrichment analysis of 174 cancer proteins predicted to be dephosphorylated by at least 4 phosphatase families. (E) A network of pathways predicted to be regulated by different cancer-associated proteins. (F) Predicted dephosphorylation sites and upstream PP families of TP53_HUMAN with GPSD were visualized by DOG 2.0.

Given that numerous signalling pathways in humans are regulated by both phosphorylation and dephosphorylation, the dysregulation of PP activity has been reported to be associated with human cancer [50]. In this study, we analysed the relationships between dephosphorylation events and cancer via our developed tool, GPSD, and explored the potential essential role of these events in tumorigenesis. First, a total of 739 cancer-related proteins collected in COSMIC [51] were downloaded, their corresponding protein sequences were used as the input for the GPSD prediction tool. The potential relationships between the dephosphorylation sites and the 12 PP groups were inferred via the high threshold. Our results revealed that 675 (91.34%) proteins were predicted to be dephosphorylated by at least one type of PP (Fig. 6A). Moreover, 214 proteins had >10 modification sites (Fig. 6B and Supplementary Table S6), suggesting that dephosphorylation might serve as a potential mechanism to reshape protein function.

Next, GO-based enrichment analyses were performed for 174 cancer proteins that had predictive modification sites of at least four PP groups. Transcription-related pathways and phosphorylation-related pathways were overrepresented, implying that the potentially important role of modification events is correlated with cancer (Fig. 6C). Furthermore, KEGG-based enrichment analyses were conducted for 174 cancer proteins, and classical cancer-related pathways were significantly enriched. Thus, our findings suggested that dephosphorylation might have a potential function in cancer-related pathways (Fig. 6D). Moreover, a network containing typical cancer-associated proteins and their related processes was constructed via enrichment analysis (Fig. 6E).

The human TP53 protein (UniProt ID: P04637), a well-studied tumour suppressor, was selected as an example to analyse PPP2CA-specific dephosphorylation sites via GPSD (Fig. 6F and Fig. 3). There are five potential modification sites in the TP53 protein, including three previously reported dephosphorylation sites, S37, S46, and T55 [52–54]. T55 phosphorylation is involved in modulating DNA binding, controlling both the activation and termination of p53-mediated transcriptional programs at different stages of the cellular DNA damage response [55]. Moreover, the dephosphorylation of S46 in TP53 may impair its apoptotic activity [56]. For S315, a predicted dephosphorylation site for PPP2CA, we carefully checked the PPI information from public data resources and reported that PPP2CA physically interacts with TP53 [57]. Moreover, S315 was identified as a dephosphorylation site specific for the PP CDC14A, and its modification modulates the function of TP53 [50]. Here, the peptide sequence, including the modification sites of S315, aligned with [ST]P, a well-characterized motif recognized by PPs in the PP2A family [50]. Therefore, on the basis of these analyses, the dephosphorylation of S315 might be regulated by PPP2CA. Collectively, our results revealed the relationship between dephosphorylation modification and human cancer and the potential mechanism involved.

## Discussion

Protein phosphorylation was first discovered in 1955 by Edwin G. Krebs and Edmond H. Fischer, who were later awarded the Nobel Prize in Physiology or Medicine in 1992 [58]. Both phosphorylation and dephosphorylation are catalysed by numerous enzymes, with PPs playing a key role in controlling the substrate specificity of dephosphorylation. Reports indicate that defective or dysregulated PP expression can contribute to cancer, highlighting the increasing importance of PPs as drug targets [7, 59]. Thus, identifying dephosphorylation sites and their corresponding upstream PPs is critical for understanding the molecular mechanisms of dephosphorylation. However, the accumulation of dephosphorylation site data has been relatively slow in recent years. In addition to traditional LTP biochemical experimental strategies, recent HTP technologies have focused primarily on a limited number of PPs, such as PP1 and PP2A [14, 17]. Another important aspect is that there are only a few experimentally identified dephosphorylation site databases, including DEPOD [35] and dbPTM [32]. Therefore, we anticipate that advancements in dephosphorylation prediction tools will positively impact the field by promoting data generation and driving progress in related areas.

In this study, we integrated 10 sequence features and 3 machine learning methods for the prediction of dephosphorylation sites (Fig. 1). From our results, it was found that each of the 10 sequence features trained by PLR, DNNs, or TNNs exhibited a considerable but differential contribution for improving the performance values of final models (Fig. 2C). Indeed, integration of DNNs, PLR, and TNNs into a hybrid learning framework further improved the accuracy for predicting general and phosphatase-specific dephosphorylation sites. Meanwhile, the current tool focuses primarily on general site prediction; the prediction of phosphatase-specific dephosphorylation sites remains underdeveloped. To address this gap, we collected 4276 experimentally identified ssPSRs from the literature and databases. In this study, PPs were manually classified into three levels on the basis of information from the iEKPD database [28]. Transfer learning and meta-learning were then applied to each PP cluster to construct the models. Finally, we implemented an online service of GPSD, which provided 2 general prediction models and 103 phosphatase-specific prediction models. In GPSD, PP clusters with ≥3 dephosphorylation sites are retained, although their reliability may be relatively low. However, including these clusters would provide more comprehensive predictions and support further experimental validation.

While GPSD is the first predictor that can broadly predict phosphatase-specific substrates and sites, it considers only the characteristics of flanking sequences around dephosphorylation sites; therefore, the prediction results need further experimental validation. Our future plans include the integration of novel computational methods into GPSD, which will be crucial for accurately predicting ssPSRs and providing valuable insights into functionally associated dephosphorylation events *in vivo*. Besides, we aim to expand the benchmark dataset to increase the number of general and phosphatase-specific dephosphorylation sites, further improving model performance and accuracy. Given the frequent crosstalk between PTMs [60, 61]—such as PRL2 dephosphorylating the tyrosine 371 site of the E3 ubiquitin ligase CBL, thereby reducing CBL-mediated ubiquitination and FLT3 degradation, which in turn enhances FLT3 signalling in leukaemia cells [62]—an improved algorithm that incorporates the relationships amongst different PTM types could significantly increase prediction accuracy. Taken together, we will continue to maintain and improve GPSD algorithm for analysing eukaryotic dephosphorylation events.

**Key Points**

- We manually curated 4393 site-specific phosphatase–substrate relationships for 3463 dephosphorylation sites occurring on phosphoserine, phosphothreonine, and phosphotyrosine residues, as well as their corresponding 106 upstream protein phosphatases.
- For the prediction of general dephosphorylation sites, we developed a hybrid learning framework by integrating 10 types of sequence features and 3 types of machine learning methods, namely, penalized logistic regression (PLR), deep neural networks (DNNs), and transformer neural networks (TNNs).
- We fine-tuned 103 individual phosphatase-specific predictors via combining transfer learning and meta-learning, and implemented an online service named GPSD for predicting phosphatase-specific dephosphorylation sites.

## Supplementary Material

Supplementary_Fig_S1_bbae694

Supplementary_Fig_S2_bbae694

Supplementary_Fig_S3_bbae694

Supplementary_Fig_S4_bbae694

Supplementary_Fig_S5_bbae694

Table_S1_bbae694

Table_S2_bbae694

Table_S3_bbae694

Table_S4_bbae694

Table_S5_bbae694

Table_S6_bbae694

Supplementary_Data-R1_bbae694

## Data Availability

All data utilized in this study are provided in the supplementary tables, as detailed in the Methods section. The source code and models used for general dephosphorylation sites prediction of our tool are freely available on GitHub (https://github.com/BioCUCKOO/GPSD).

## References

[1] Wilson LJ , Linley A, Hammond DE. et al. New perspectives, opportunities, and challenges in exploring the human protein kinome. Cancer Res 2018;78:15–29. 10.1158/0008-5472.CAN-17-2291. 29254998

[2] Manning G , Whyte DB, Martinez R. et al. The protein kinase complement of the human genome. Science 2002;298:1912–34. 10.1126/science.1075762. 12471243

[3] Ubersax JA , Ferrell JE, Jr. Mechanisms of specificity in protein phosphorylation. Nat Rev Mol Cell Biol 2007;8:530–41. 10.1038/nrm2203. 17585314

[4] Hunter T . Protein kinases and phosphatases: the yin and yang of protein phosphorylation and signaling. Cell 1995;80:225–36. 10.1016/0092-8674(95)90405-0. 7834742

[5] Ardito F , Giuliani M, Perrone D. et al. The crucial role of protein phosphorylation in cell signaling and its use as targeted therapy (review). Int J Mol Med 2017;40:271–80. 10.3892/ijmm.2017.3036. 28656226 PMC5500920

[6] Klumpp S , Krieglstein J. Phosphorylation and dephosphorylation of histidine residues in proteins. Eur J Biochem 2002;269:1067–71. 10.1046/j.1432-1033.2002.02755.x. 11856347

[7] Stanford SM , Bottini N. Targeting protein phosphatases in cancer immunotherapy and autoimmune disorders. Nat Rev Drug Discov 2023;22:273–94. 10.1038/s41573-022-00618-w. 36693907 PMC9872771

[8] Guo M , Li Z, Gu M. et al. Targeting phosphatases: from molecule design to clinical trials. Eur J Med Chem 2024;264:116031. 10.1016/j.ejmech.2023.116031. 38101039

[9] Junttila MR , Li SP, Westermarck J. Phosphatase-mediated crosstalk between MAPK signaling pathways in the regulation of cell survival. FASEB J 2008;22:954–65. 10.1096/fj.06-7859rev. 18039929

[10] Shi Y . Serine/threonine phosphatases: mechanism through structure. Cell 2009;139:468–84. 10.1016/j.cell.2009.10.006. 19879837

[11] Roskoski R, Jr . A historical overview of protein kinases and their targeted small molecule inhibitors. Pharmacol Res 2015;100:1–23. 10.1016/j.phrs.2015.07.010. 26207888

[12] Yu ZH , Zhang ZY. Regulatory mechanisms and novel therapeutic targeting strategies for protein tyrosine phosphatases. Chem Rev 2018;118:1069–91. 10.1021/acs.chemrev.7b00105. 28541680 PMC5812791

[13] Jong CJ , Merrill RA, Wilkerson EM. et al. Reduction of protein phosphatase 2A (PP2A) complexity reveals cellular functions and dephosphorylation motifs of the PP2A/B'δ holoenzyme. J Biol Chem 2020;295:5654–68. 10.1074/jbc.RA119.011270. 32156701 PMC7186168

[14] Hoermann B , Kokot T, Helm D. et al. Dissecting the sequence determinants for dephosphorylation by the catalytic subunits of phosphatases PP1 and PP2A. Nat Commun 2020;11:3583. 10.1038/s41467-020-17334-x. 32681005 PMC7367873

[15] Eguchi A , Olsen JV. Phosphoproteomic investigation of targets of protein phosphatases in EGFR signaling. Sci Rep 2024;14:7908. 10.1038/s41598-024-58619-1. 38575675 PMC10995159

[16] Won S , Incontro S, Li Y. et al. The STEP(61) interactome reveals subunit-specific AMPA receptor binding and synaptic regulation. Proc Natl Acad Sci U S A 2019;116:8028–37. 10.1073/pnas.1900878116. 30936304 PMC6475416

[17] Hein JB , Nguyen HT, Garvanska DH. et al. Phosphatase specificity principles uncovered byMRBLE:Dephos and global substrate identification. Mol Syst Biol 2023;19:e11782. 10.15252/msb.202311782. 37916966 PMC10698503

[18] Kruse T , Gnosa SP, Nasa I. et al. Mechanisms of site-specific dephosphorylation and kinase opposition imposed by PP2A regulatory subunits. EMBO J 2020;39:e103695. 10.15252/embj.2019103695. 32400009 PMC7327492

[19] Cundell MJ , Hutter LH, Nunes Bastos R. et al. A PP2A-B55 recognition signal controls substrate dephosphorylation kinetics during mitotic exit. J Cell Biol 2016;214:539–54. 10.1083/jcb.201606033. 27551054 PMC5004449

[20] Wu Z , Lu M, Li T. Prediction of substrate sites for protein phosphatases 1B, SHP-1, and SHP-2 based on sequence features. Amino Acids 2014;46:1919–28. 10.1007/s00726-014-1739-6. 24760585

[21] Wang X , Yan R, Song J. DephosSite: a machine learning approach for discovering phosphotase-specific dephosphorylation sites. Sci Rep 2016;6:23510. 10.1038/srep23510. 27002216 PMC4802303

[22] Jia C , He W, Zou Q. DephosSitePred: a high accuracy predictor for protein dephosphorylation sites. Comb Chem High Throughput Screen 2017;20:153–7. 10.2174/1386207319666161228155636. 28031011

[23] Chaudhari M , Thapa N, Ismail H. et al. DTL-DephosSite: deep transfer learning based approach to predict dephosphorylation sites. Front Cell Dev Biol 2021;9:662983. 10.3389/fcell.2021.662983. 34249915 PMC8264445

[24] Chen M , Zhang W, Gou Y. et al. GPS 6.0: an updated server for prediction of kinase-specific phosphorylation sites in proteins. Nucleic Acids Res 2023;51:W243–w250. 10.1093/nar/gkad383. 37158278 PMC10320111

[25] Vaswani A , Shazeer N, Parmar N. et al. Attention Is all You Need, Advances in Neural Information Processing Systems 30 (Nips 2017), Curran Associates Inc., 57 Morehouse Lane, Red Hook. NY, United States, Ulrike von Luxburg, 2017, 30.

[26] Kenton JDM-WC , Toutanova LK. BERT: pre-training of deep bidirectional transformers for language understanding. In: Proceedings of naacL-HLT, p. 2. Minneapolis, Minnesota, Association for Computational Linguistics, Anastassia Loukina, 2019.

[27] Radford A . Improving language understanding by generative pre-training. 2018.

[28] Guo Y , Peng D, Zhou J. et al. iEKPD 2.0: an update with rich annotations for eukaryotic protein kinases, protein phosphatases and proteins containing phosphoprotein-binding domains. Nucleic Acids Res 2019;47:D344–d350. 10.1093/nar/gky1063. 30380109 PMC6324023

[29] Finn C , Abbeel P, Levine S. Model-agnostic meta-learning for fast adaptation of deep networks. In: International Conference on Machine Learning, p. 1126-35. PMLR, Sydney NSW Australia, Doina Precup, 2017.

[30] Qin J , Huang X, Gou S. et al. Ketogenic diet reshapes cancer metabolism through lysine β-hydroxybutyrylation. Nat Metab 2024;6:1505–28. 10.1038/s42255-024-01093-w. 39134903

[31] Lin S , Wang C, Zhou J. et al. EPSD: a well-annotated data resource of protein phosphorylation sites in eukaryotes. Brief Bioinform 2021;22:298–307. 10.1093/bib/bbz169. 32008039

[32] Huang KY , Lee TY, Kao HJ. et al. dbPTM in 2019: exploring disease association and cross-talk of post-translational modifications. Nucleic Acids Res 2019;47:D298–d308. 10.1093/nar/gky1074. 30418626 PMC6323979

[33] Hornbeck PV , Kornhauser JM, Latham V. et al. 15 years of PhosphoSitePlus®: integrating post-translationally modified sites, disease variants and isoforms. Nucleic Acids Res 2019;47:D433–d441. 10.1093/nar/gky1159. 30445427 PMC6324072

[34] Chen Z , Zhao P, Li C. et al. iLearnPlus: a comprehensive and automated machine-learning platform for nucleic acid and protein sequence analysis, prediction and visualization. Nucleic Acids Res 2021;49:e60. 10.1093/nar/gkab122. 33660783 PMC8191785

[35] Damle NP , Köhn M. The human DEPhOsphorylation database DEPOD: 2019 update. Database (Oxford) 2019, baz133. 10.1093/database/baz133. PMC6911163 31836896

[36] Lundberg SM , Erion G, Chen H. et al. From local explanations to global understanding with explainable AI for trees. Nat Mach Intell 2020;2:56–67. 10.1038/s42256-019-0138-9. 32607472 PMC7326367

[37] Oughtred R , Stark C, Breitkreutz BJ. et al. The BioGRID interaction database: 2019 update. Nucleic Acids Res 2019;47:D529–d541. 10.1093/nar/gky1079. 30476227 PMC6324058

[38] Maddelein D , Colaert N, Buchanan I. et al. The iceLogo web server and SOAP service for determining protein consensus sequences. Nucleic Acids Res 2015;43:W543–6. 10.1093/nar/gkv385. 25897125 PMC4489316

[39] Dosztányi Z , Csizmok V, Tompa P. et al. IUPred: web server for the prediction of intrinsically unstructured regions of proteins based on estimated energy content. Bioinformatics 2005;21:3433–4. 10.1093/bioinformatics/bti541. 15955779

[40] Wang J , Wang Z, Yu T. et al. Crystal structure of a PP2A B56-BubR1 complex and its implications for PP2A substrate recruitment and localization. Protein Cell 2016;7:516–26. 10.1007/s13238-016-0283-4. 27350047 PMC4930772

[41] Nguyen H , Kettenbach AN. Substrate and phosphorylation site selection by phosphoprotein phosphatases. Trends Biochem Sci 2023;48:713–25. 10.1016/j.tibs.2023.04.004. 37173206 PMC10523993

[42] Terrak M , Kerff F, Langsetmo K. et al. Structural basis of protein phosphatase 1 regulation. Nature 2004;429:780–4. 10.1038/nature02582. 15164081

[43] Drewes G , Mandelkow EM, Baumann K. et al. Dephosphorylation of tau protein and Alzheimer paired helical filaments by calcineurin and phosphatase-2A. FEBS Lett 1993;336:425–32. 10.1016/0014-5793(93)80850-T. 8282105

[44] McCloy RA , Parker BL, Rogers S. et al. Global phosphoproteomic mapping of early mitotic exit in human cells identifies novel substrate dephosphorylation motifs. Mol Cell Proteomics 2015;14:2194–212. 10.1074/mcp.M114.046938. 26055452 PMC4528247

[45] Wrighton KH , Willis D, Long J. et al. Small C-terminal domain phosphatases dephosphorylate the regulatory linker regions of Smad2 and Smad3 to enhance transforming growth factor-beta signaling. J Biol Chem 2006;281:38365–75. 10.1074/jbc.M607246200. 17035229

[46] Liu X , Dong M, Yao Y. et al. A tyrosine phosphoproteome analysis approach enabled by selective dephosphorylation with protein tyrosine phosphatase. Anal Chem 2022;94:4155–64. 10.1021/acs.analchem.1c03704. 35239328

[47] Asante-Appiah E , Ball K, Bateman K. et al. The YRD motif is a major determinant of substrate and inhibitor specificity in T-cell protein-tyrosine phosphatase. J Biol Chem 2001;276:26036–43. 10.1074/jbc.M011697200. 11352902

[48] Espanel X , Huguenin-Reggiani M, Hooft van Huijsduijnen R. et al. The SPOT technique as a tool for studying protein tyrosine phosphatase substrate specificities. Protein Sci 2002;11:2326–34. 10.1110/ps.0213402. 12237455 PMC2373693

[49] Amanchy R , Periaswamy B, Mathivanan S. et al. A curated compendium of phosphorylation motifs. Nat Biotechnol 2007;25:285–6. 10.1038/nbt0307-285. 17344875

[50] Paulsen MT , Starks AM, Derheimer FA. et al. The p53-targeting human phosphatase hCdc14A interacts with the Cdk1/cyclin B complex and is differentially expressed in human cancers. Mol Cancer 2006;5:25. 10.1186/1476-4598-5-25. 16784539 PMC1524803

[51] Forbes SA , Beare D, Gunasekaran P. et al. COSMIC: exploring the world's knowledge of somatic mutations in human cancer. Nucleic Acids Res 2015;43:D805–11. 10.1093/nar/gku1075. 25355519 PMC4383913

[52] Dohoney KM , Guillerm C, Whiteford C. et al. Phosphorylation of p53 at serine 37 is important for transcriptional activity and regulation in response to DNA damage. Oncogene 2004;23:49–57. 10.1038/sj.onc.1207005. 14712210

[53] Mi J , Bolesta E, Brautigan DL. et al. PP2A regulates ionizing radiation-induced apoptosis through Ser46 phosphorylation of p53. Mol Cancer Ther 2009;8:135–40. 10.1158/1535-7163.MCT-08-0457. 19139122

[54] Li HH , Cai X, Shouse GP. et al. A specific PP2A regulatory subunit, B56gamma, mediates DNA damage-induced dephosphorylation of p53 at Thr55. EMBO J 2007;26:402–11. 10.1038/sj.emboj.7601519. 17245430 PMC1783465

[55] Sun X , Dyson HJ, Wright PE. A phosphorylation-dependent switch in the disordered p53 transactivation domain regulates DNA binding. Proc Natl Acad Sci U S A 2021;118:e2021456118. 10.1073/pnas.2021456118. PMC7817127 33443163

[56] Garufi A , D'Orazi G. High glucose dephosphorylates serine 46 and inhibits p53 apoptotic activity. J Exp Clin Cancer Res 2014;33:79. 10.1186/s13046-014-0079-4. 25260780 PMC4181716

[57] Shouse GP , Nobumori Y, Panowicz MJ. et al. ATM-mediated phosphorylation activates the tumor-suppressive function of B56γ-PP2A. Oncogene 2011;30:3755–65. 10.1038/onc.2011.95. 21460856

[58] Fischer EH , Krebs EG. Conversion of phosphorylase b to phosphorylase a in muscle extracts. J Biol Chem 1955;216:121–32. 10.1016/S0021-9258(19)52289-X. 13252012

[59] Vainonen JP , Momeny M, Westermarck J. Druggable cancer phosphatases. Sci Transl Med 2021;13:13. 10.1126/scitranslmed.abe2967. 33827975

[60] Yu F , Wu Y, Xie Q. Precise protein post-translational modifications modulate ABI5 activity. Trends Plant Sci 2015;20:569–75. 10.1016/j.tplants.2015.05.004. 26044742

[61] Vu LD , Gevaert K, De Smet I. Protein language: post-translational modifications talking to each other. Trends Plant Sci 2018;23:1068–80. 10.1016/j.tplants.2018.09.004. 30279071

[62] Chen H , Bai Y, Kobayashi M. et al. PRL2 phosphatase enhances oncogenic FLT3 signaling via dephosphorylation of the E3 ubiquitin ligase CBL at tyrosine 371. Blood 2023;141:244–59. 10.1182/blood.2022016580. 36206490 PMC9936309

