# Supplementary figures and images for: GPSD: a hybrid learning framework for the prediction of phosphatase-specific dephosphorylation sites
Source: Brief Bioinform. 2025 Jan 2;26(1):bbae694. doi: 10.1093/bib/bbae694 (PMC11695897; doi:10.1093/bib/bbae694)

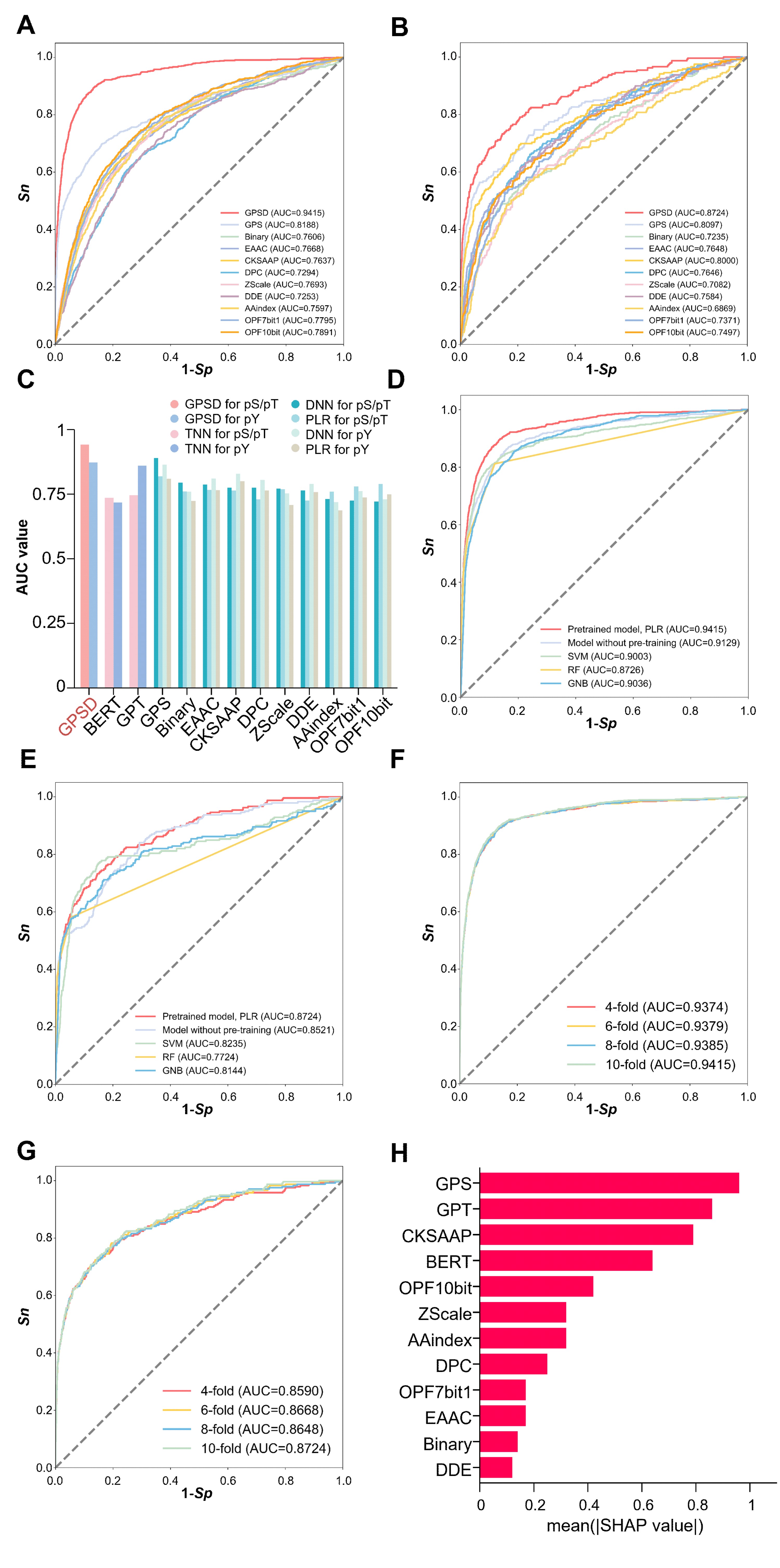

Supplement: Supplementary_Fig_S1_bbae694 [file supplementary_fig_s1_bbae694.jpeg]

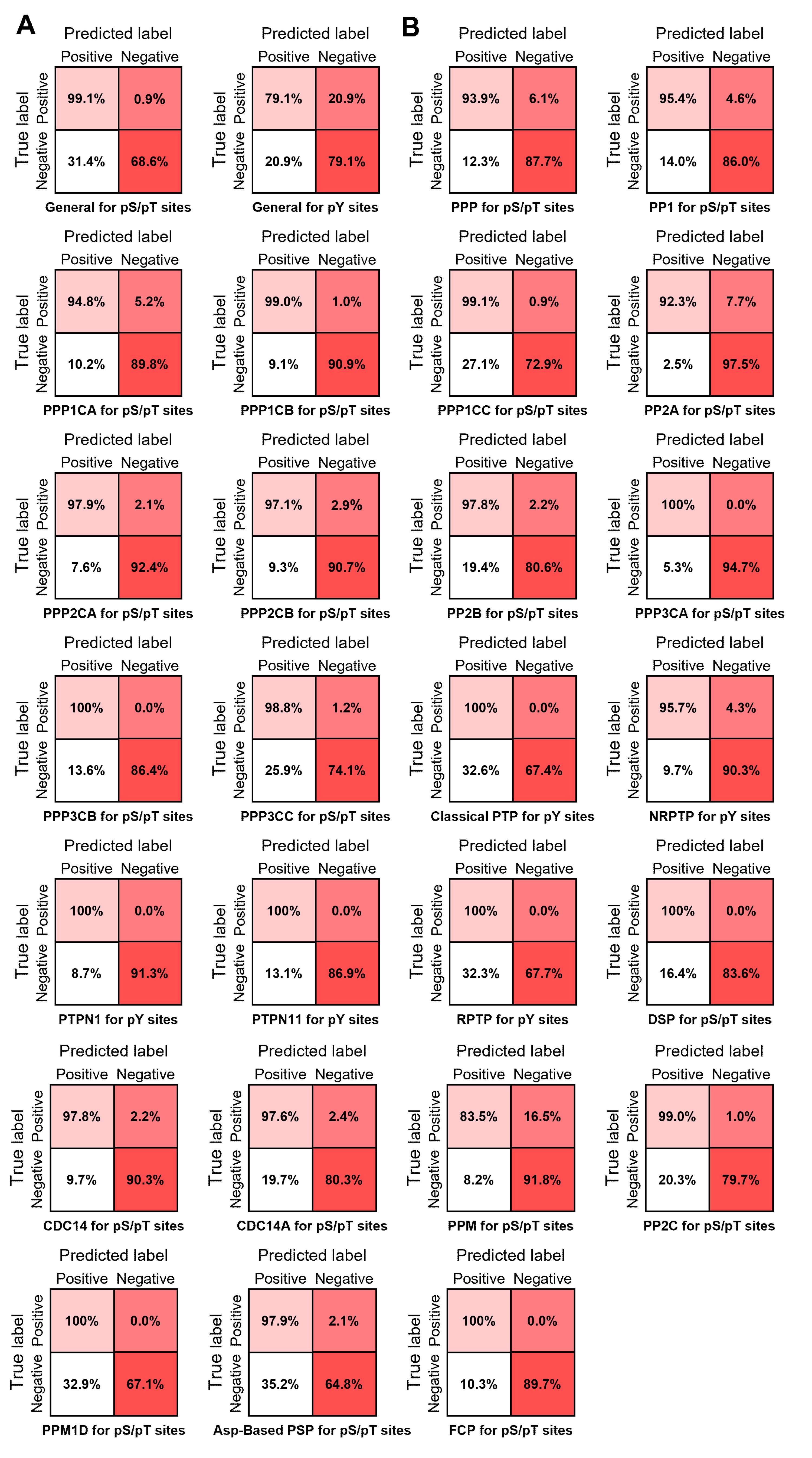

Supplement: Supplementary_Fig_S2_bbae694 [file supplementary_fig_s2_bbae694.jpeg]

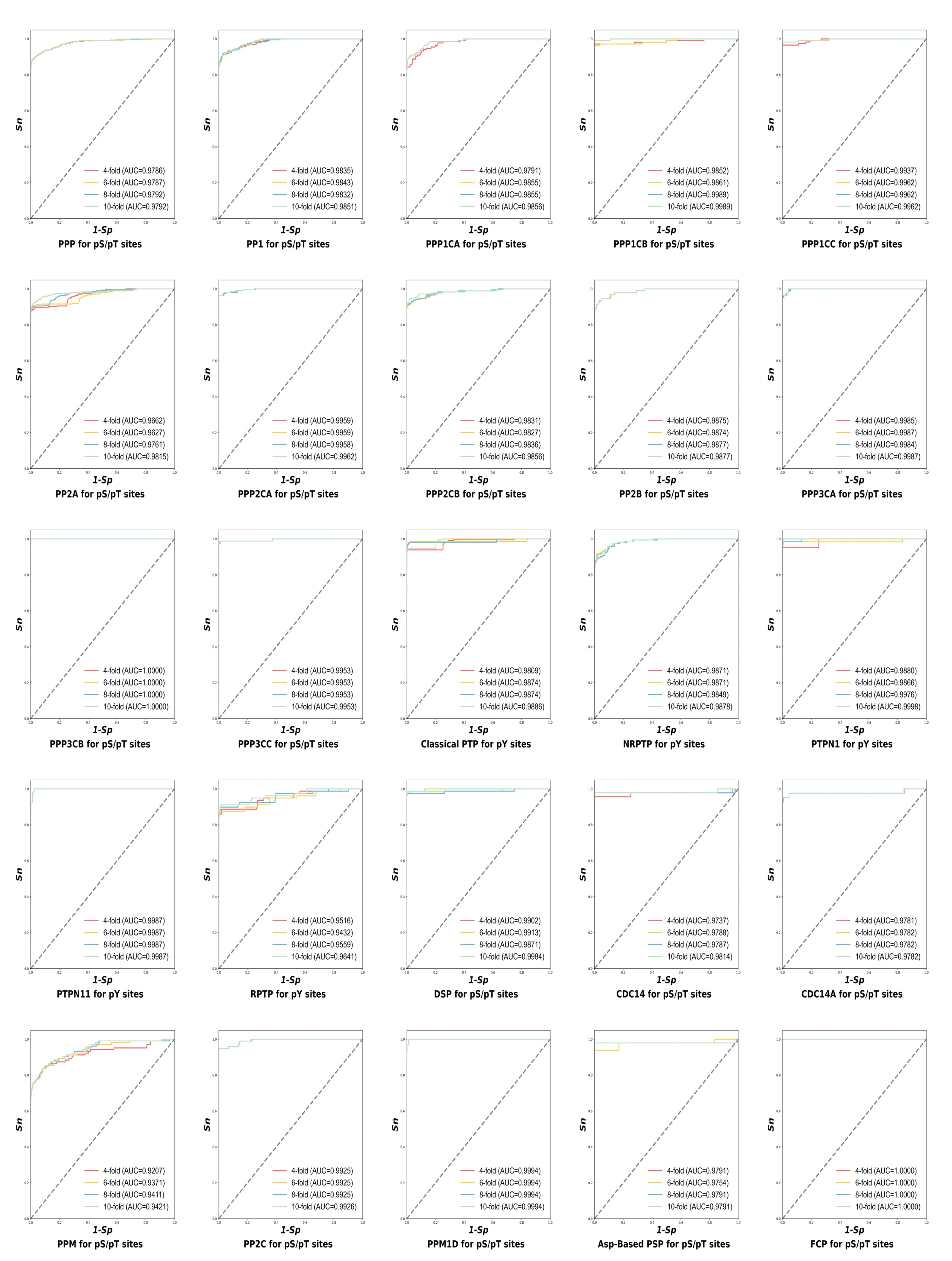

Supplement: Supplementary_Fig_S3_bbae694 [file supplementary_fig_s3_bbae694.jpeg]

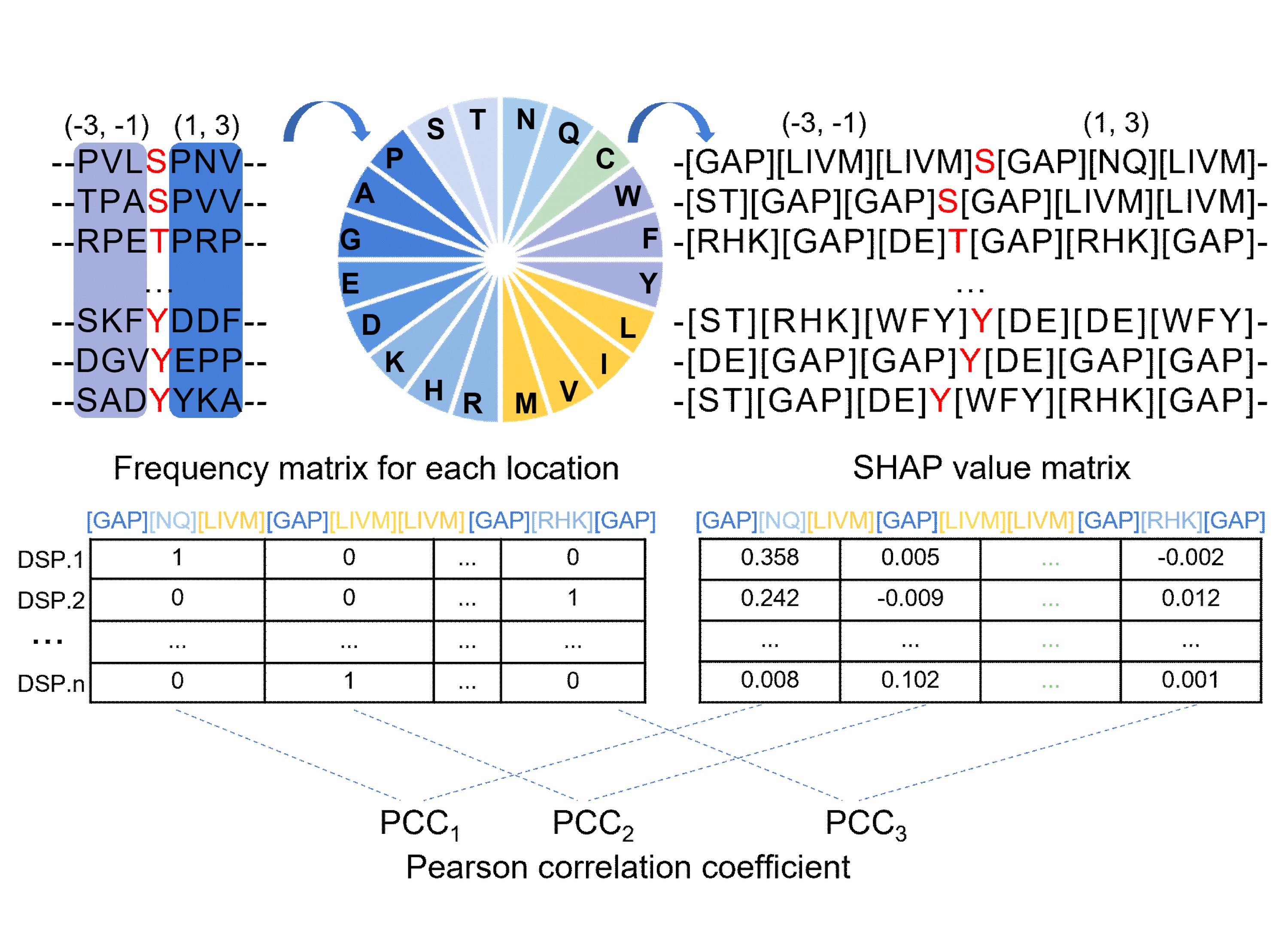

Supplement: Supplementary_Fig_S4_bbae694 [file supplementary_fig_s4_bbae694.jpeg]

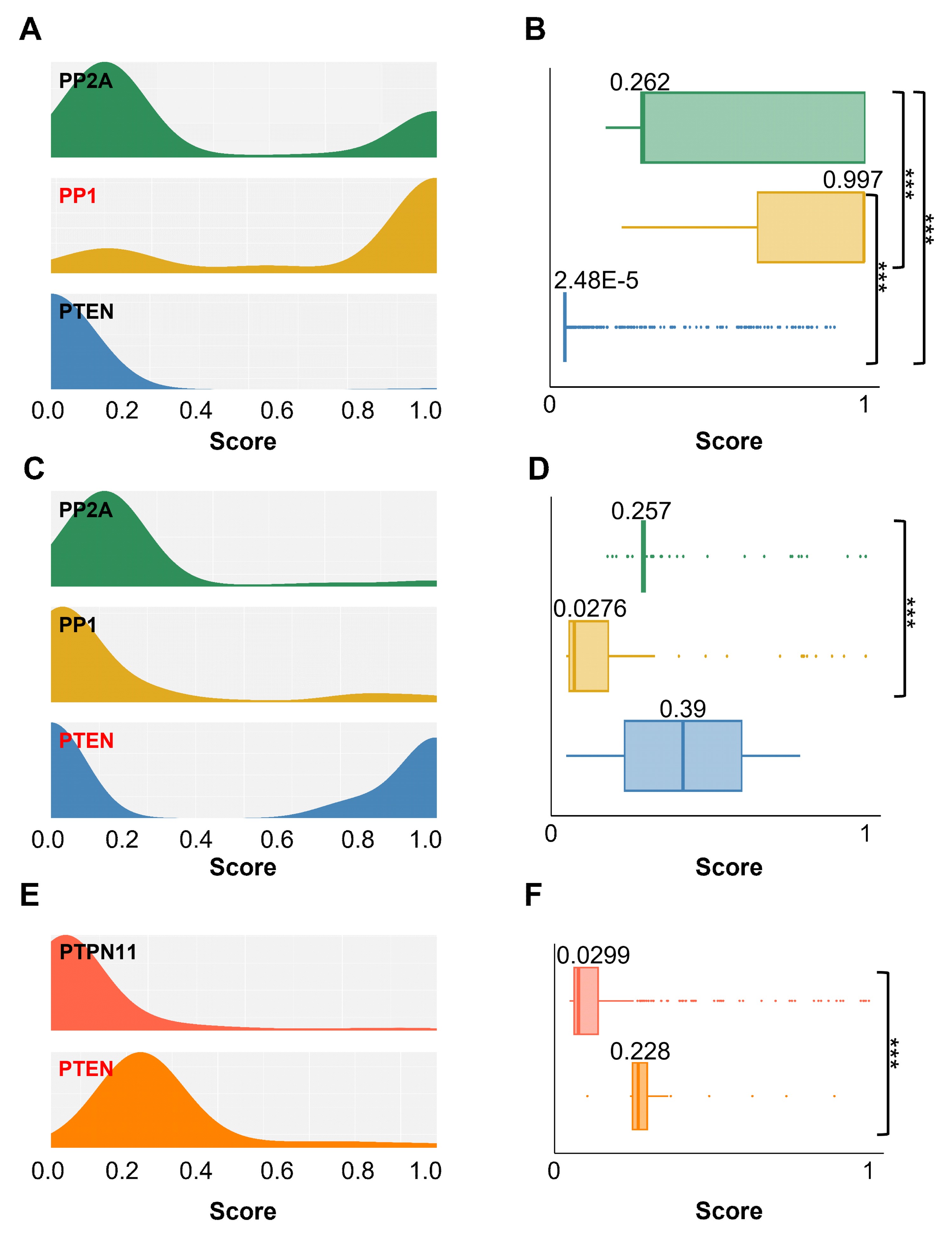

Supplement: Supplementary_Fig_S5_bbae694 [file supplementary_fig_s5_bbae694.jpeg]
