# Supplementary material for: GPSD: a hybrid learning framework for the prediction of phosphatase-specific dephosphorylation sites
Source: Brief Bioinform. 2025 Jan 2;26(1):bbae694. doi: 10.1093/bib/bbae694 (PMC11695897; doi:10.1093/bib/bbae694)
Supplement: Supplementary_Data-R1_bbae694 [file supplementary_data-r1_bbae694.docx]

Supplementary Data:

GPSD: A hybrid learning framework for the prediction of phosphatase-specific dephosphorylation sites

Cheng Han^1,†^, Shanshan Fu^1,†^, Miaomiao Chen^1^, Yujie Gou^1^, Dan Liu^1^, Chi Zhang^1^, Xinhe Huang^1^, Leming Xiao^1^, Miaoying Zhao^1^, Jiayi Zhang^1^, Qiang Xiao^2^, Di Peng^1,*^, Yu Xue^1,*^

^1^Department of Bioinformatics and Systems Biology, MOE Key Laboratory of Molecular Biophysics, Hubei Bioinformatics and Molecular Imaging Key Laboratory, Center for Artificial Intelligence Biology, College of Life Science and Technology, Huazhong University of Science and Technology, 430074 Wuhan, Hubei, China.

^2^School of Artificial Intelligence and Automation, Huazhong University of Science and Technology, 430074 Wuhan, Hubei, China.

*Corresponding authors: Yu Xue, Department of Bioinformatics and Systems Biology, MOE Key Laboratory of Molecular Biophysics, Hubei Bioinformatics and Molecular Imaging Key Laboratory, Center for Artificial Intelligence Biology, College of Life Science and Technology, Huazhong University of Science and Technology, 430074 Wuhan, Hubei, China. Email: xueyu@hust.edu.cn; Di Peng, Department of Bioinformatics and Systems Biology, MOE Key Laboratory of Molecular Biophysics, Hubei Bioinformatics and Molecular Imaging Key Laboratory, Center for Artificial Intelligence Biology, College of Life Science and Technology, Huazhong University of Science and Technology, 430074 Wuhan, Hubei, China. Email: [pengdi@hust.edu.cn](mailto:pengdi@hust.edu.cn)

^†^The first two authors contributed equally to this work.

Supplementary methods

**Data collection and preparation**

First, we downloaded 1,616,804 experimentally identified phosphorylation sites (p-sites) in 209,326 eukaryotic proteins from EPSD [[1](#_ENREF_1)]. Also, we obtained 86,084 additional p-sites from two public databases, including dbPTM [[2](#_ENREF_2)] and PhosphoSitePlus [[3](#_ENREF_3)]. A widely used clustering program, CD-HIT [[4](#_ENREF_4)], was adopted to classify this dataset into different clusters with a threshold of 40% sequence similarity. To avoid homologous redundancy, only one representative sequence in each cluster was extracted into the training data. As previously described [[5](#_ENREF_5)], both positive and negative PSP(30,30) items were extracted. For the S/T or Y residues located near the N- or C-terminus of the protein sequences, one or multiple characters ‘*’ were added to complement the PSP(30,30) items. After removing the redundant PSP(30,30) items, 561,416 nonredundant PSP(30,30) items were obtained from 82,468 proteins as a positive dataset, and the same number of negative datasets were generated by randomly selecting from the negative PSP(30,30) items. This dataset was used for pretraining two general phosphorylation models for S/T and Y sites, respectively.

For the dephosphorylation site dataset, we downloaded 764 known site-specific phosphatase–substrate relationships (ssPSRs) from 347 human dephosphorylation sites in DEPOD [[6](#_ENREF_6)], and obtained 133 additional dephosphorylation sites from dbPTM [[2](#_ENREF_2)]. Using a number of keywords, such as "dephosphorylate", "dephosphorylated" and "dephosphorylation", we searched the literature published by PubMed up to April 2024, and manually collected 1,208 dephosphorylation sites on 663 proteins. From high-throughput experiments, we obtained 2,373 ssPSRs from 2,304 dephosphorylation sites [[7-11](#_ENREF_7)]. These data focused on proteins associated with mitotic exit processes regulated by phosphatases PP1 and PP2A, possibly due to the important role of PP1 and PP2A in mitosis [[9](#_ENREF_9), [10](#_ENREF_10)]. In total, we obtained 4,393 ssPSRs for 3,463 dephosphorylation sites of 1,833 proteins and 106 protein phosphatases (PPs) (Supplementary Table S1). The training data contained 3,304 positive sites in 1,765 proteins published before April 1, 2023, and 159 positive sites published after April 1, 2023 were taken as an independent dataset for testing. Then, dephosphorylatable PSP(30,30) items around experimentally identified dephosphorylation sites were taken as positive data, whereas PSP(30,30) items around other pS/pT or pY residues were regarded as negative data. This dataset was used for fine-tuning general dephosphorylation models, as well as further fine-tuning phosphatase-specific models.

Before fine-tuning for phosphatase-specific models, we carefully re-checked each ssPSR, replaced phosphatases from other species with human orthologs, and matched all phosphatases with iEKPD (https://iekpd.biocuckoo.org/) [[12](#_ENREF_12)]. Only phosphatase clusters with ≥ 3 dephosphorylation sites were retained for further training. Then, the remaining 4,267 known ssPSRs were subsequently classified into different phosphatase clusters at the group, family, and single phosphatase levels, For each phosphatase cluster, the negative data were negative dephosphorylatable PSP(30,30) corresponding to the general dephosphorylation model. Finally, 103 phosphatase clusters with ≥ 3 known sites were included.

**Sequence feature encoding scheme**

Similar to our previous studies [[5](#_ENREF_5), [13](#_ENREF_13), [14](#_ENREF_14)], each PSP(30,30) in the training dataset was first encoded by each of the 10 types of sequence features: 1) GPS, which encodes the position-weighted similarity of amino acids into a matrix [[5](#_ENREF_5)]; 2) Binary, which is also termed one-hot encoding; 3) CKSAAP, which is the composition of *k*-spaced amino acid pairs; in this study, *k*=3; 4) DDE, which is the dipeptide deviation from the expected mean feature vector and is constructed by computing three parameters, dipeptide composition, theoretical mean, and theoretical variance; 5) DPC, which is the dipeptide composition; 6) EAAC, which has an enhanced amino acid composition; in this study, we chose 5 as the sliding window size; 7) AAindex, which is a database that contains 566 amino acid indices of physicochemical properties; in this study, we specifically selected the following eight indices: 'ANDN920101', 'ARGP820101', 'ARGP820102', 'ARGP820103', 'BEGF750101', 'BEGF750102', 'BEGF750103', and 'BHAR880101'; 8) OPF_10bit (OPF10), which classifies amino acids into 10 groups on the basis of their physicochemical properties; 9) OPF_7bit type 1 (OPF1), which is similar to OPF_10bit and classifies amino acids into 7 groups; and 10) ZScale, which includes five physicochemical descriptor variables, was utilized to characterize amino acid variation in neurotensin (NT) at Positions 8, 9, and 11 [[15](#_ENREF_15)].

Except the GPS feature, the last 9 features were chosen from iLearnPlus [[16](#_ENREF_16)] according to the receiver operating characteristic (ROC) corresponding to the AUC trained through a penalized logistic regression (PLR) approach.

**Implementation of deep neural networks (DNNs) and transformer neural networks (TNNs)**

The DNNs consisted of four layers, including an input layer, two hidden layers, and an output layer. Each layer comprises a specific number of computational units called neurons, and the dropout method was used to randomly discard nodes from the two hidden layers if the accuracy increased. For each neuron in the input layer and hidden layers, a received vector *x* was transformed by the rectified linear unit (ReLU) as the activation function, which was defined as follows:

$$ReLU\left( x \right)=\left\{ \begin{aligned} x, &x\geq0 \\ 0, &x<0 \end{aligned} \right.$$

The output layer contains two sigmoid neurons adopted to calculate the final score for a given PSP(30,30) *y*, defined as:

$$score\left( y \right)=sigmoid\left( y \right)=\frac{1}{1+e^{-y}}$$

Using the one-hot encoding, we further took the two TNNs of BERT and GPT, to learn the contextual information [[17-19](#_ENREF_17)]. For either BERT or GPT, PSP(30,30) items were encoded with both positional and token features. The final embedding *E* of PSP(30,30) was defined as follows:

$$E=\mathrm{Embedding}_{\mathrm{token}}+\mathrm{Embedding}_{\mathrm{pos}\mathrm{ition}}$$

In the BERT-based model, the transformer encoder module consists of multi-head attention mechanism and feed-forward neural network (FFN). The multi-head attention mechanism includes 4 individual attention heads that operate in parallel. For each head, the input feature *E* is multiplied by three linear layers, *W_q_*, *W_k_*, and *W_v_*, to produce the Query (*Q*), key (*K*), and value (*V*) matrices. The attention was then computed as follows:

$$\mathrm{Attent}\mathrm{ion}\left( Q,K,V \right)=\mathrm{softmax} \left( \frac{QK^{T}}{\sqrt{d_{k}}} \right)V$$

Next, the attention heads were concatenated, and the final multi-head attention layer was represented as:

$$\mathrm{MultiHead} \left( Q,K,V \right)=Concat\left( \mathrm{head}_{1},\ldots,\mathrm{head}_{4} \right){W_{o}}$$

Here, *W_q_*, *W_k_*, *W_v_* and *W_o_* were the weight matrices that would be learnt during training.

The output of the multi-head attention layer was processed by the layer normalization step before entering the FFN. The FFN consists of two linear transformations with a Gaussian Error Linear Unit (GELU) activation in between. The GELU activation function is defined as follows:

$$GELU\left( x \right)=x\cdot\Phi\left( x \right)$$

where Φ(x) represents the cumulative distribution function of a standard Gaussian distribution.

The final scores from the output layer were calculated using the softmax function to evaluate the probability of each PSP(30,30) item. The softmax function was defined as follows:

$$\mathrm{Softmax}(x_{i})=\frac{\exp(x_{i})}{\underset{j}{\sum}\exp(x_{j})}$$

where *x_i_* represents the score for each class.

In the GPT-based model, the transformer decoder also includes multi-head attention and an FFN, with a causal mask applied to enforce autoregressive training. This mask prevents the model from seeing future tokens during training, enforcing an autoregressive property essential for language generation tasks. The causal mask *M* is defined as follows:

$$M_{ij}=\left\{ \begin{aligned} 0, &i\geq j \\ -\infty, &i<j \end{aligned} \right.$$

The output of the multi-head attention layer in GPT-based model is similarly processed by a layer normalization step before entering the FFN. The FFN consists of two linear transformations with a ReLU activation in between. The final scores from the output layer were also calculated via the softmax function.

**Performance evaluation measurements**

To evaluate the accuracy of the GPSD, four commonly used measurements, including sensitivity (*Sn*), specificity (*Sp*), accuracy (*Ac*), and the Matthew correlation coefficient (*MCC*), were calculated as follows:

$$Sn= \frac{TP}{TP+FN}$$

$$Sp= \frac{TN}{TN+FP}$$

$$Ac= \frac{TP+TN}{TP+FP+TN+FN}$$

$$MCC= \frac{\left( TP\times TN \right)-(FN\times FP)}{\sqrt{\left( TP+FN \right)\times(TN+FP)\times(TP+FP)\times(TN+FN)}}$$

As we implemented 10 features in GPSD, 10-fold cross-validations were performed for each feature, whereas 4-, 6-, 8-, and 10-fold cross-validations were used to evaluate the accuracy and robustness of the final predictor. (Fig. S1F, G). An independent test dataset of 159 positive dephosphorylation sites was used to compare the performance of our tool with that of other existing tools. For predicting phosphatase-specific sites, the robustness of models with ≥ 30 sites was tested with 10-fold cross-validations and confusion matrix with threshold of *Sp* = 90%, and leave-one-out (LOO) validations were performed for other models with < 30 sites. For each model, the ROC curve was plotted on the basis of the *Sn* and 1-*Sp* scores. The AUC values, along with other relevant measurements, were subsequently calculated and are provided in Supplementary Table S3.

**Model interpretation**

To investigate which motif sequences play an important role in the training of the dephosphorylation model, we counted the frequency of upstream and downstream short peptides centred on the dephosphorylation sites. We truncated the dephosphorylation residue flanked by 3 upstream residues and 3 downstream residues to form a short peptide, and the resulting dephosphorylatable PSP(3,3) was statistically analysed.

According to the published literature [[20](#_ENREF_20)], we classified the twenty amino acids into 8 categories for more efficient analysis of the characteristics of peptides on the basis of their properties, including aromatic [WFY], hydrophobic [LIVM], acidic [DE], basic [RHK], small [GAP], small and polar [ST], polar [NQ] and C. For each position, the sparse frequency matrix was calculated with dephosphorylatable PSP(3,3) as the row and all amino acid combinations as the columns (Supplementary Fig. S4). A 4-layer DNN model was constructed to predict the frequency and dephosphorylation state of short peptides. SHapley Additive exPlanation (SHAP) [[21](#_ENREF_21)], a machine learning method used to assess the contributions of different features, was applied to measure peptide contributions. Important peptides were identified on the basis of two criteria: first, we calculated Pearson correlation coefficients (PCCs) between peptide frequencies and their corresponding SHAP values; second, we used the normalized average SHAP values to provide an overall assessment of peptide contributions to dephosphorylation states (Supplementary Fig. S4). In this case, we screened for peptides with |PCC|>0.2 and a normalized score>0.15 (Fig. 4A-D) as important peptides adjacent to dephosphorylation residue candidates. All the PCCs and scores of the peptides are provided in Supplementary Table S5.

**The hypergeometric test**

The hypergeometric test was adopted for the enrichment analysis of proteins containing predicted significant motifs in the dephosphorylatable PSP(30,30). For each experimentally identified dephosphorylation motif pattern t, we defined the following:

*N* = number of all peptides in the dephosphorylation benchmark dataset.

*n* = number of positive dephosphorylation peptides in the benchmark dataset.

*M* = number of peptides conforming to motif pattern t.

*m* = number of positive dephosphorylation peptides conforming to motif pattern *t*.

The enrichment ratio (E-ratio) of *t* was then computed, and the *p* value was calculated with the hypergeometric distribution as follows:

$$E-ratio= {\frac{m}{M}}/{\frac{n}{N}}$$

$p-value= \sum_{m^{'}=m}^{n} \frac{\binom{M}{m^{'}}\binom{N-M}{n-m^{'}}}{\binom{N}{n}}$ (E-ratio ≥ 1), or

$p-value= \sum_{m^{'}=0}^{m} \frac{\binom{M}{m^{'}}\binom{N-M}{n-m^{'}}}{\binom{N}{n}}$ (E-ratio < 1)

The hypergeometric test was also adopted for GO-based and KEGG-based enrichment analyses of cancer genes predicted to be dephosphorylated by phosphatase groups. A total of 739 cancer-related proteins were downloaded from the Cancer Gene Census (CGC) in the Catalogue of Somatic Mutations in Cancer (COSMIC) (https://cancer.sanger.ac.uk/census, v100) [[22](#_ENREF_22)]. The GO annotation files (released on 17 June 2024) [[23](#_ENREF_23)] were downloaded from the Gene Ontology Resource (<http://geneontology.org/>). The KEGG annotation files were downloaded from the Kyoto Encyclopedia of Genes and Genomes (https://www.kegg.jp/) [[24](#_ENREF_24)].

**Phosphatase-specific dephosphorylation site prediction**

The general models for predicting dephosphorylation sites were transferred to phosphatase-specific data, and the network of each phosphatase group was fine-tuned. To demonstrate the specificity and efficiency of our model, we first predicted the dephosphorylation sites at each group level. Specifically, 12 group-specific dephosphorylation models were used to cross-predict the specific datasets corresponding to 12 phosphatase groups (Fig. 5A). To further verify model specificity, we applied the pS/pT-specific phosphatase models PP2A and PP1, as well as the dual-specificity phosphatase model PTEN, to predict the PP1-, PP2A-, and PTEN-specific datasets and compared the specificity of each phosphatase predictor (Fig. 5B, C, and Supplementary Fig. S5A-D). Similarly, the pY-specific model PTPN11 and the dual-specificity phosphatase model PTEN were used to predict their corresponding datasets (Fig. 5D, E, and Supplementary Fig. S5E, F).

**Data visualization**

The sequence logo was generated by uploading all positive dephosphorylatable PSP(30,30) that matched the motif patterns to the web service WebLogo (http://weblogo.berkeley.edu/logo.cgi) [[25](#_ENREF_25)]. The heatmap was generated via the previously developed tool HemI [[26](#_ENREF_26)]. Cytoscape [[27](#_ENREF_27)] was used to visualize the network of cancer-related proteins and their regulatory pathways. In addition, the functional domain and predicted dephosphorylation sites of TP53 were illustrated via DOG 2.0 [[28](#_ENREF_28)].

**Web server implementation**

The web server consists of a front end implemented with PHP 7.0.33 and JQuery 1.4.4. The back end, which implements the GPSD algorithm framework, was written in Python 3.8 and uses PHP to render tabular prediction results. Chart visualizations are created via JavaScript libraries, whereas 3D structures are retrieved from the PDB database and visualized with 3Dmol.js (http://3dmol.csb.pitt.edu/) [[29](#_ENREF_29)]. For each pS/pT-specific predictor, we selected three thresholds, namely, high, medium and low, on the basis of *Sp* values of 98%, 94% and 90%, respectively, whereas *Sp* values of 95%, 90% and 85%, respectively, were selected for the pY-specific predictors. In the web server of GPSD, the high threshold was chosen as the default configuration. The GPSD web server was tested across three common operating systems—Linux, macOS, and Windows—using the appropriate browsers for each. The results confirm that our predictive tool is compatible with various computer systems and browsers. For reference, the version numbers of each operating system and browser can be viewed at https://gpsd.biocuckoo.cn/userguide.php.

**Supplementary References**

1. Lin S, Wang C, Zhou J et al. EPSD: a well-annotated data resource of protein phosphorylation sites in eukaryotes, Brief Bioinform 2021;22:298-307.

2. Huang KY, Lee TY, Kao HJ et al. dbPTM in 2019: exploring disease association and cross-talk of post-translational modifications, Nucleic Acids Res 2019;47:D298-d308.

3. Hornbeck PV, Kornhauser JM, Latham V et al. 15 years of PhosphoSitePlus®: integrating post-translationally modified sites, disease variants and isoforms, Nucleic Acids Res 2019;47:D433-d441.

4. Fu L, Niu B, Zhu Z et al. CD-HIT: accelerated for clustering the next-generation sequencing data, Bioinformatics 2012;28:3150-3152.

5. Chen M, Zhang W, Gou Y et al. GPS 6.0: an updated server for prediction of kinase-specific phosphorylation sites in proteins, Nucleic Acids Res 2023;51:W243-w250.

6. Damle NP, Köhn M. The human DEPhOsphorylation Database DEPOD: 2019 update, Database (Oxford) 2019;2019.

7. Hein JB, Nguyen HT, Garvanska DH et al. Phosphatase specificity principles uncovered by MRBLE:Dephos and global substrate identification, Mol Syst Biol 2023;19:e11782.

8. Kruse T, Gnosa SP, Nasa I et al. Mechanisms of site-specific dephosphorylation and kinase opposition imposed by PP2A regulatory subunits, Embo j 2020;39:e103695.

9. Hoermann B, Kokot T, Helm D et al. Dissecting the sequence determinants for dephosphorylation by the catalytic subunits of phosphatases PP1 and PP2A, Nat Commun 2020;11:3583.

10. Cundell MJ, Hutter LH, Nunes Bastos R et al. A PP2A-B55 recognition signal controls substrate dephosphorylation kinetics during mitotic exit, J Cell Biol 2016;214:539-554.

11. Jong CJ, Merrill RA, Wilkerson EM et al. Reduction of protein phosphatase 2A (PP2A) complexity reveals cellular functions and dephosphorylation motifs of the PP2A/B'δ holoenzyme, J Biol Chem 2020;295:5654-5668.

12. Guo Y, Peng D, Zhou J et al. iEKPD 2.0: an update with rich annotations for eukaryotic protein kinases, protein phosphatases and proteins containing phosphoprotein-binding domains, Nucleic Acids Res 2019;47:D344-d350.

13. Ning W, Xu H, Jiang P et al. HybridSucc: A Hybrid-learning Architecture for General and Species-specific Succinylation Site Prediction, Genomics Proteomics Bioinformatics 2020;18:194-207.

14. Wang C, Tan X, Tang D et al. GPS-Uber: a hybrid-learning framework for prediction of general and E3-specific lysine ubiquitination sites, Brief Bioinform 2022;23.

15. Sandberg M, Eriksson L, Jonsson J et al. New chemical descriptors relevant for the design of biologically active peptides. A multivariate characterization of 87 amino acids, J Med Chem 1998;41:2481-2491.

16. Chen Z, Zhao P, Li C et al. iLearnPlus: a comprehensive and automated machine-learning platform for nucleic acid and protein sequence analysis, prediction and visualization, Nucleic Acids Res 2021;49:e60.

17. Vaswani A, Shazeer N, Parmar N et al. Attention Is All You Need, Advances in Neural Information Processing Systems 30 (Nips 2017) 2017;30.

18. Kenton JDM-WC, Toutanova LK. Bert: Pre-training of deep bidirectional transformers for language understanding. In: Proceedings of naacL-HLT. 2019, p. 2. Minneapolis, Minnesota.

19. Radford A. Improving language understanding by generative pre-training 2018.

20. Kim Y, Sidney J, Pinilla C et al. Derivation of an amino acid similarity matrix for peptide: MHC binding and its application as a Bayesian prior, BMC Bioinformatics 2009;10:394.

21. Lundberg SM, Erion G, Chen H et al. From Local Explanations to Global Understanding with Explainable AI for Trees, Nat Mach Intell 2020;2:56-67.

22. Forbes SA, Beare D, Gunasekaran P et al. COSMIC: exploring the world's knowledge of somatic mutations in human cancer, Nucleic Acids Res 2015;43:D805-811.

23. Huntley RP, Sawford T, Mutowo-Meullenet P et al. The GOA database: gene Ontology annotation updates for 2015, Nucleic Acids Res 2015;43:D1057-1063.

24. Kanehisa M, Goto S. KEGG: kyoto encyclopedia of genes and genomes, Nucleic Acids Res 2000;28:27-30.

25. Crooks GE, Hon G, Chandonia JM et al. WebLogo: a sequence logo generator, Genome Res 2004;14:1188-1190.

26. Ning W, Wei Y, Gao L et al. HemI 2.0: an online service for heatmap illustration, Nucleic Acids Res 2022;50:W405-w411.

27. Shannon P, Markiel A, Ozier O et al. Cytoscape: a software environment for integrated models of biomolecular interaction networks, Genome Res 2003;13:2498-2504.

28. Ren J, Wen L, Gao X et al. DOG 1.0: illustrator of protein domain structures, Cell Res 2009;19:271-273.

29. Rego N, Koes D. 3Dmol.js: molecular visualization with WebGL, Bioinformatics 2015;31:1322-1324.

**Supplementary Figures**

**Supplementary Figure S****1**. Performance evaluation of GPSD. (**A**) Performance evaluation of the predictors of pS/pT dephosphorylation sites via various algorithms and features. The 10 models were individually trained via the PLR algorithm and each of 10 sequence features. GPSD models were constructed by integrating 10 features and 3 machine learning methods. (**B**) Performance evaluation of the predictors of pY dephosphorylation sites via various algorithms and features. Similar to the pS/pT dephosphorylation site predictors, the ROC curves and AUC values of 11 models were presented. (**C**) For each of 10 sequence features, the AUC values of predictive models trained with DNNs, PLR, and TNNs, were calculated for the general dephosphorylation site predictor. (**D**) Performance comparison of the predictive model for pS/pT dephosphorylation sites with or without the pretrained models of phosphorylation, and performance of feature integration utilizing SVM, RF, and GNB. (**E**) Performance comparison of the predictive model for pY dephosphorylation sites with or without the pretrained models of phosphorylation, and performance of feature integration utilizing SVM, RF, and GNB. (**F**) *n*-fold cross-validations for the models of predicting pS/pT dephosphorylation site. For the evaluation of the predictive models, 4-, 6-, 8-, and 10-fold cross-validations were used, and the ROC curves and AUC values are presented. (**G**) *n*-fold cross-validations for the predictors of analysing pY dephosphorylation site. Similar procedure was conducted as described in (F). (**H**) Contribution of 10 types of sequence features and contextual information captured by TNNs to the predictors of pY dephosphorylation site using SHAP method.

**Supplementary Figure S2**. The confusion matrices for models trained with ≥ 30 known sites. (**A**) The confusion matrices of 2 general predictive models were analyzed using a threshold of *Sp* = 90%. (**B**) The confusion matrices of 25 phosphatase-specific predictors were analyzed using a threshold of *Sp* = 90%.

**Supplementary Figure S3**. Performance evaluation of 25 phosphatase-specific predictive models trained with ≥ 30 known sites using *n*-fold cross-validations. For the evaluation of the predictive models, 4-, 6-, 8-, and 10-fold cross-validations were used, and the ROC curves and AUC values are presented.

**Supplementary Figure S4**. The motif analysis method calculates PCCs between peptide frequency matrices and their corresponding SHAP values and reveals that the functional motif sequence may play a significant role in dephosphorylation.

**Supplementary Figure S5**. Specificity analysis of phosphatase dephosphorylation site predictors. (**A, B**) Density (**A**) and box (**B**) charts showing the distributions of pS/pT dephosphorylation site predictors specific for PP2A, PP1 and PTEN in the PP1 dataset. (**C, D**) Density (**C**) and box (**D**) charts showing the distributions of pS/pT dephosphorylation site predictors specific for PP2A, PP1 and PTEN in the PTEN dataset. (**E, F**) Density (**E**) and box (**F**) charts showing the distributions of pY dephosphorylation site predictors specific for PTPN11 and PTEN in the PTEN dataset.
